# Co-targeting RNA Polymerases IV and V Promotes Efficient *De Novo* DNA Methylation in *Arabidopsis*

**DOI:** 10.1016/j.cell.2019.01.029

**Published:** 2019-02-21

**Authors:** Javier Gallego-Bartolomé, Wanlu Liu, Peggy Hsuanyu Kuo, Suhua Feng, Basudev Ghoshal, Jason Gardiner, Jenny Miao-Chi Zhao, Soo Young Park, Joanne Chory, Steven E. Jacobsen

**Affiliations:** 1 Department of Molecular, Cell and Developmental Biology, University of California at Los Angeles, Los Angeles, CA 90095, USA; 2 Eli & Edythe Broad Center of Regenerative Medicine & Stem Cell Research, University of California at Los Angeles, Los Angeles, CA 90095, USA; 3 Howard Hughes Medical Institute, La Jolla, CA 92037, USA; 4 The Salk Institute, La Jolla, CA 92037, USA; 5 Howard Hughes Medical Institute, University of California at Los Angeles, Los Angeles, CA 90095, USA

## Abstract

The RNA-directed DNA methylation (RdDM) pathway in plants controls gene expression via cytosine DNA methylation. The ability to manipulate RdDM would shed light on the mechanisms and applications of DNA methylation to control gene expression. Here, we identified diverse RdDM proteins that are capable of targeting methylation and silencing in *Arabidopsis* when tethered to an artificial zinc finger (ZF-RdDM). We studied their order of action within the RdDM pathway by testing their ability to target methylation in different mutants. We also evaluated ectopic siRNA biogenesis, RNA polymerase V (Pol V) recruitment, targeted DNA methylation, and gene-expression changes at thousands of ZF-RdDM targets. We found that co-targeting both arms of the RdDM pathway, siRNA biogenesis and Pol V recruitment, dramatically enhanced targeted methylation. This work defines how RdDM components establish DNA methylation and enables new strategies for epigenetic gene regulation via targeted DNA methylation.

## Highlights

• A panel of RdDM factors can target DNA methylation when fused to an artificial ZF
• ZF-RdDM fusions in different mutant backgrounds reveal RdDM hierarchy of action
• MORC6 can target DNA methylation
• Co-targeting of Pol IV and Pol V synergistically enhances DNA methylation targeting

## Graphical Abstract

**Figure:**
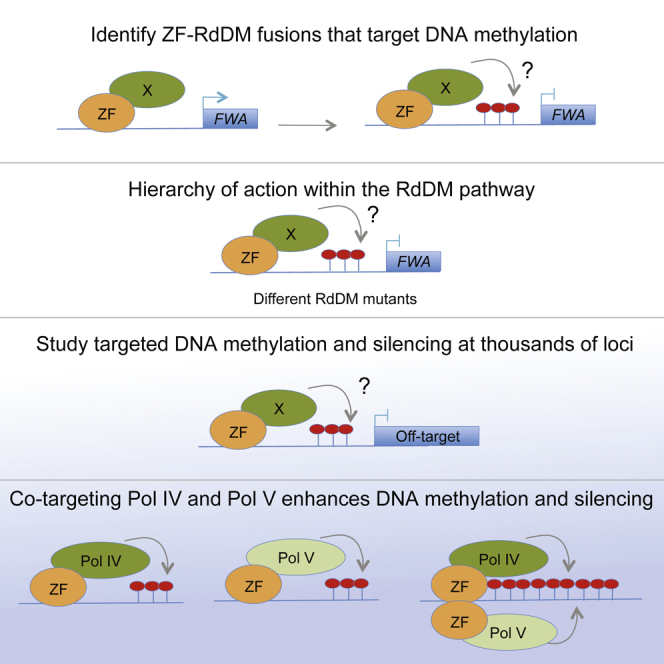

## Teaser

Comprehensive investigation into the genetic pathway of RNA-directed DNA methylation in *Arabidopsis* defines epistatic relationships and allows for efficient manipulation of DNA methylation to specifically modify developmental phenotypes.

## Introduction

Cytosine DNA methylation is a key epigenetic mark involved in the silencing of transposable elements (TEs) and genes in eukaryotes. Improving our knowledge of the pathways that trigger methylation in plants is critical, not only for understanding how methylation is established naturally, but also for creating DNA-methylation-targeting tools that can be used to establish novel epigenetic alleles of important plant genes. Plant genomes are methylated within three sequence contexts: CG, CHG, or CHH (where H is A, T, C) (Law and Jacobsen, 2010). Methylation establishment requires the plant-specific RNA-directed DNA methylation (RdDM) pathway, which can be divided into two major arms and acts via the *de novo* DNA methyltransferase DOMAINS REARRANGED METHYLTRANSFERASE 2 (DRM2) (Law and Jacobsen, 2010).

**Figure S1:**
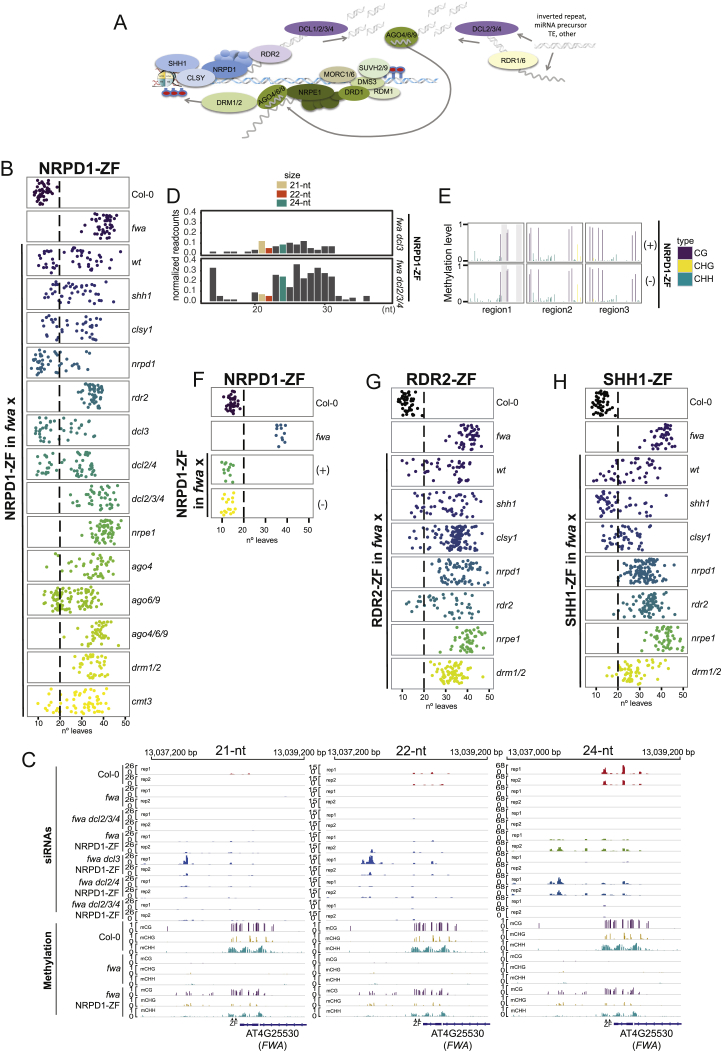
NRPD1, RDR2, and SHH1 Targeted Methylation, Related Figure 1 (A) Model of RNA-directed DNA methylation pathway. (B) Flowering time of Col-0 and *fwa* untransformed plants, as well as NRPD1-ZF T1 lines in different mutant backgrounds introgressed into *fwa* mutant. (C) Screenshots of 21-nt, 22-nt and 24-nt siRNAs accumulation over the *FWA* promoter in two untransformed Col-0, *fwa* and *fwa dcl2 dcl3 dcl4* (*fwa dcl2/3/4*) plants as well as in two biological replicates of NRPD1-ZF T2 lines in different mutant backgrounds introgressed into the *fwa* mutant. Methylation levels at different context (CG, CHG and CHH, where H is A, T, C) over the *FWA* promoter in Col-0, *fwa* and one representative NRPD1-ZF line in *fwa* background are shown. ZF binding sites are indicated with triangles. (D) Barplot of normalized readcounts for different sizes of sRNAs over the 200 bp covering the ZF binding sites in the *FWA* promoter in NRPD1-ZF lines in *fwa dcl3* and *fwa dcl2 dcl3 dcl4 (dcl2/3/4)* backgrounds. 21-nt, 22-nt and 24-nt siRNAs are marked in different colors. Counts from two biological replicates were merged for each genotype. (E) CG, CHG and CHH DNA methylation levels measured by BS-PCR-seq over the *FWA* promoter in NRPD1-ZF T3 plants that contain (+) or have segregated the transgene away (-). (F) Flowering time of Col-0 and *fwa* untransformed plants as well as NRPD1-ZF T3 plants that contain (+) or have segregated the transgene away (-). (G) Flowering time of Col-0 and *fwa* untransformed plants as well as RDR2-ZF T1 lines in different mutant backgrounds introgressed into *fwa* mutant. (H) Flowering time of Col-0 and *fwa* untransformed plants as well as SHH1-ZF T1 lines in different mutant backgrounds introgressed into *fwa* mutant. Although we observed a small number of SHH1-ZF T1 plants in a *drm1 drm2* mutant background that showed early flowering, these lines were all late flowering in the T2 suggesting that the T1 early flowering phenotype observed was caused by something other than *FWA* silencing such as stress.

In the RdDM “arm 1,” RNA polymerase IV (Pol IV) generates transcripts (P4-RNAs) that are converted into double-stranded RNAs (dsRNA) by RNA-DEPENDENT RNA POLYMERASE 2 (RDR2) and subsequently processed into 24-nt siRNAs by DICER-LIKE 3 (DCL3) (Kuo et al., 2017, Matzke et al., 2015) (Figure S1A). In the absence of DCL3, other DICER-LIKE proteins—DCL1, DCL2, and DCL4—can process P4-RNAs into 21-nt or 22-nt small interfering RNAs (siRNAs) that trigger *de novo* methylation by RdDM (Bond and Baulcombe, 2015, Henderson et al., 2006). Mutations in *NRPD1*, the Pol IV catalytic subunit, lead to a virtually complete loss of 24-nt siRNAs genome wide (Matzke et al., 2015) and a strong DNA-methylation loss at RdDM sites (Stroud et al., 2013). Pol IV accessory proteins include the CLASSY SNF2-related putative chromatin remodeler family (CLSY) involved in global Pol IV recruitment (Zhou et al., 2018), and SAWADEE HOMEODOMAIN HOMOLOG 1 (SHH1), which binds the repressive histone mark H3K9 methylation associated with DNA methylation and is required for Pol IV recruitment at a subset of RdDM sites (Law et al., 2013). siRNAs for RdDM can be alternatively generated from other RNAs, like viral or Pol-II dependent, in “non-canonical RdDM” (Cuerda-Gil and Slotkin, 2016). These RNAs can be processed into dsRNAs by RDR1 and RDR6 and subsequently cleaved into siRNAs by different DCL proteins (Cuerda-Gil and Slotkin, 2016). Subsequently, siRNAs are loaded into ARGONAUTE 4 (AGO4) or its homologs, AGO6 and AGO9 (Matzke et al., 2015).

In the RdDM arm 2, RNA Pol V, together with a number of accessory proteins, generates longer non-coding RNAs at target loci (Böhmdorfer et al., 2016, Liu et al., 2018, Matzke et al., 2015) (Figure S1A). The DNA-methylation reader proteins SU(VAR)3-9 homologs SUVH2 and SUVH9, as well as the DDR complex consisting of RNA-DIRECTED DNA METHYLATION 1 (RDM1), DEFECTIVE IN MERISTEM SILENCING 3 (DMS3), and DEFECTIVE IN RNA-DIRECTED DNA METHYLATION 1 (DRD1), are required globally for Pol V occupancy on chromatin (Johnson et al., 2014, Liu et al., 2014, Zhong et al., 2012). siRNA-loaded AGO4 interacts with Pol V through its C-terminal domain (El-Shami et al., 2007, Li et al., 2006), and it is thought that homologous pairing between siRNAs and Pol V RNAs leads to AGO4-mediated DRM2 recruitment (Zhong et al., 2014), although many aspects of these molecular details remain unknown.

Other factors implicated in RdDM include the microrchidia (MORC) ATPases, MORC1 and MORC6, that act as heterodimers to mediate gene silencing (Matzke et al., 2015, Moissiard et al., 2012, Moissiard et al., 2014). Unlike RdDM mutants, *morc* mutants show reactivation of many methylated regions without a corresponding DNA-methylation loss and thus appear to act downstream of DNA methylation at most loci. However, *morc* mutants do show a loss of DNA methylation at a small subset of RdDM loci that are also transcriptionally derepressed in *morc* mutants (Harris et al., 2016, Matzke et al., 2015). In addition, physical interactions between MORC1 and MORC6 with the RdDM proteins SUVH2, SUVH9, IDN2, and DMS3 have been reported (Jing et al., 2016, Liu et al., 2014, Liu et al., 2016, Lorković et al., 2012, Matzke et al., 2015), although the functional relevance of these interactions and the specific role of MORCs in RdDM remain unclear.

The imprinted gene *FLOWERING WAGENINGEN* (*FWA*) (Soppe et al., 2000) is normally repressed by promoter DNA methylation in wild-type plants. Loss of *FWA* methylation creates heritable *fwa* epialleles showing ectopic *FWA* expression and a late flowering phenotype (Soppe et al., 2000). We previously showed that tethering the RdDM component SUVH9 to an artificial zinc finger (ZF) that targets the *FWA* promoter can reintroduce heritable DNA methylation, restoring *FWA* repression and early flowering (Johnson et al., 2014).

Here, we show that ZF fusions with many other RdDM components can also promote DNA methylation at *FWA*, as well as at thousands of additional loci targeted by this ZF. Importantly, Pol IV and Pol V co-targeting synergistically enhanced targeted methylation, revealing that siRNA biogenesis and recruitment of the DNA-methylation machinery are largely independent and both important for efficient methylation targeting. We also utilized ZF-RdDM fusions to dissect the hierarchy of action of RdDM components, providing unprecedented mechanistic insight into *de novo* DNA methylation. These findings provide a framework for the study and manipulation of DNA-methylation patterns in plants.

## Results

### Novel ZF Fusion Proteins that Promote *FWA* Methylation

**Figure S2:**
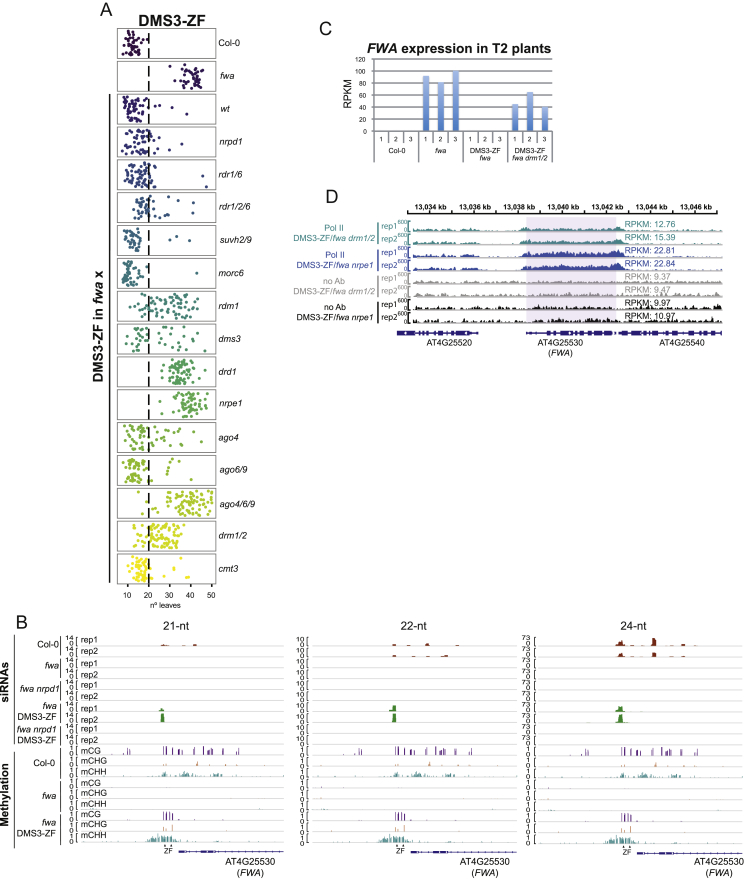
DMS3 Targeted Methylation, Related Figure 2 (A) Flowering time of Col-0 and *fwa* untransformed plants as well as DMS3-ZF T1 lines in different mutant backgrounds introgressed into *fwa* mutant. (B) Screenshot of 21-nt, 22-nt and 24-nt siRNAs accumulation over the *FWA* promoter in two untransformed Col-0, *fwa* and *fwa nrpd1* plants as well as in two representative T2 lines expressing DMS3-ZF in *fwa* and *fwa nrpd1* backgrounds. Methylation levels at different context (CG, CHG and CHH) over the *FWA* promoter in Col-0, *fwa* and one representative DMS3-ZF line are shown. ZF binding sites are indicated with triangles. (C) *FWA* expression measured by RNA-seq in three independent pools of seedlings from untransformed Col-0 and *fwa* mutants and pools of seedlings from three independent T2 lines expressing DMS3-ZF in the *fwa* and *fwa drm1 drm2 (fwa drm1/2)* backgrounds. (D) Screenshot of two biological replicates of Pol II Ser5 and no antibody control (no Ab) ChIP-seq signal over the *FWA* region in DMS3-ZF lines *in fwa drm1/2* and *fwa nrpe1* backgrounds. RPKM values from the *FWA* transcribed region are presented for each track.

**Figure S3:**
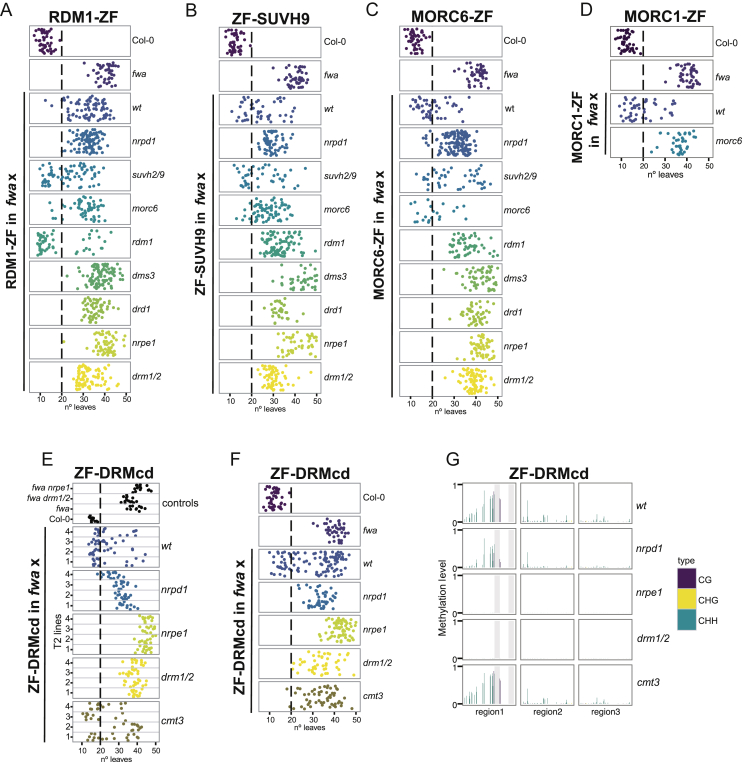
RDM-1, SUVH9-, and MORC-Mediated Targeted Methylation, Related Figure 3 Flowering time of Col-0 and *fwa* untransformed plants as well as T1 plants of (A) RDM1-ZF, (B) ZF-SUVH9, (C) MORC6-ZF and (D) MORC1-ZF in different mutant backgrounds introgressed into *fwa* mutant. (E) Flowering time of 4 representative ZF-DRMcd T2 lines in different mutant backgrounds. wt corresponds to single *fwa* mutant and the rest of named mutants are in *fwa* background. Flowering time of Col-0, *fwa, fwa nrpe1 and fwa drm1 drm2 (fwa drm1/2)* controls is shown. (F) Flowering time of Col-0 and *fwa* untransformed plants as well as ZF-DRMcd T1 lines in different mutant backgrounds introgressed into *fwa* mutant. (G) CG, CHG and CHH DNA methylation levels over the *FWA* promoter in representative ZF-DRMcd T2 lines measured by BS-PCR-seq.

We utilized the targeting approach previously described in Johnson et al. (2014) to test RdDM components for their ability to promote DNA methylation when fused to ZF. When transformed into the unmethylated *fwa* background, 9 different fusion proteins restored an early-flowering phenotype indicative of DNA methylation and silencing of *FWA* in T1 plants. These included components of the first (NRPD1, RDR2, and SHH1; Figures S1B, S1G, and S1H) and second (DMS3, RDM1, and SUVH9; Figures S2A, S3A, and S3B) arms of the RdDM pathway, MORC6 and MORC1 (Figures S3C and S3D) and the catalytic domain of the tobacco DRM2 methyltransferase (DRMcd) (Figure S3F).

**Figure 1:**
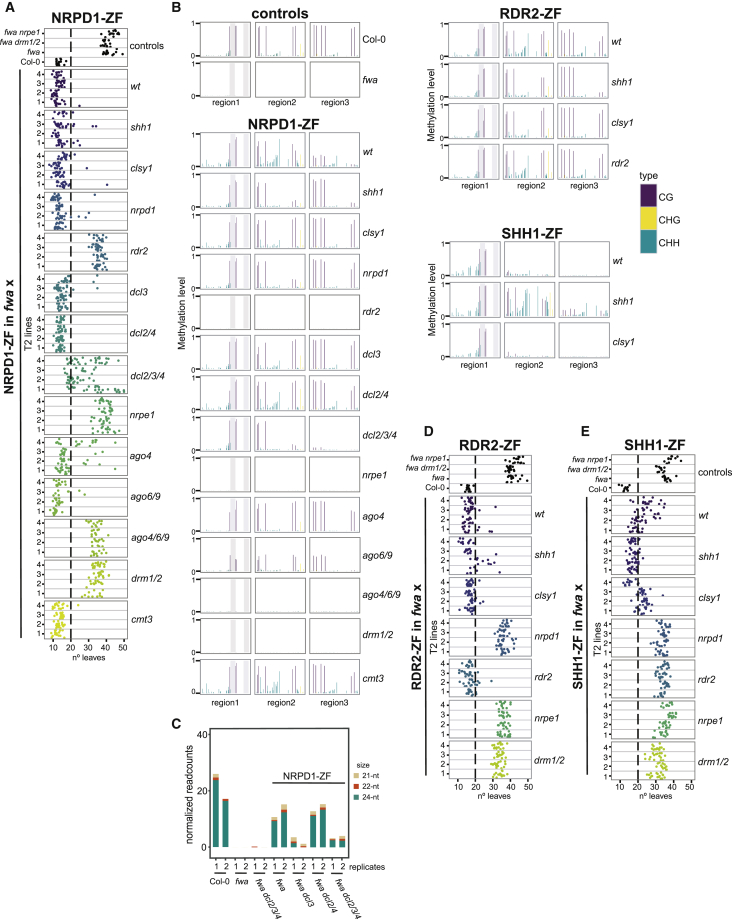
NRPD1, RDR2, and SHH1 Targeted Methylation (A) Flowering time of control lines and four representative NRPD1-ZF T2 lines in wild-type or different mutants that had been introgressed into the *fwa* background. (B) CG, CHG, and CHH DNA methylation levels over the *FWA* promoter measured by bisulfite (BS)-PCR-seq in Col-0 and *fwa* controls, as well as NRPD1-ZF, RDR2-ZF, and SHH1-ZF T2 lines in wild-type and different mutants introgressed into *fwa*. Gray vertical lines indicate the ZF binding sites. (C) Normalized siRNA abundance over the 200 bp covering the ZF binding sites in the *FWA* promoter in two biological replicates of different genotypes. (D) Flowering time of control lines and four representative RDR2-ZF T2 lines in different mutant backgrounds. (E) Flowering time of control lines and four representative SHH1-ZF T2 lines in different mutant backgrounds. See also Figure S1.

**Figure 2:**
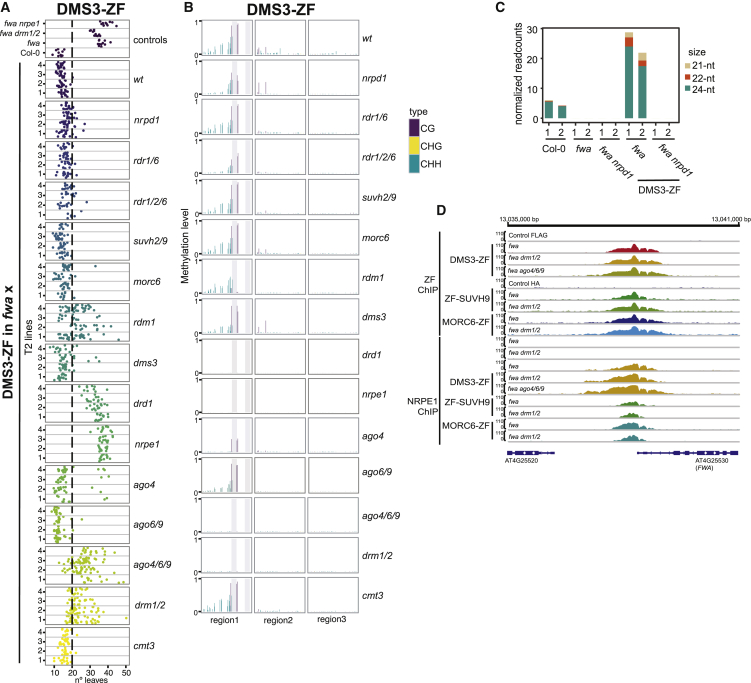
DMS3 Targeted Methylation (A) Flowering time of control lines and four representative DMS3-ZF T2 lines in wild-type or different mutants that had been introgressed into the *fwa* background. (B) CG, CHG, and CHH DNA-methylation levels over the *FWA* promoter measured by BS-PCR-seq in representative DMS3-ZF T2 lines in wild-type or different mutants introgressed into *fwa*. Gray vertical lines indicate the ZF binding sites. (C) Normalized siRNA abundance over the 200 bp covering the ZF binding sites in the *FWA* promoter in two biological replicates of untransformed controls and two independent DMS3-ZF lines in *fwa* and *fwa nrpd1* backgrounds. (D) ZF and NRPE1 ChIP-seq signals over the *FWA* promoter in untransformed controls and the listed ZF-RdDM transgenic lines transformed into different genotypes. See also Figure S2.

**Figure 3:**
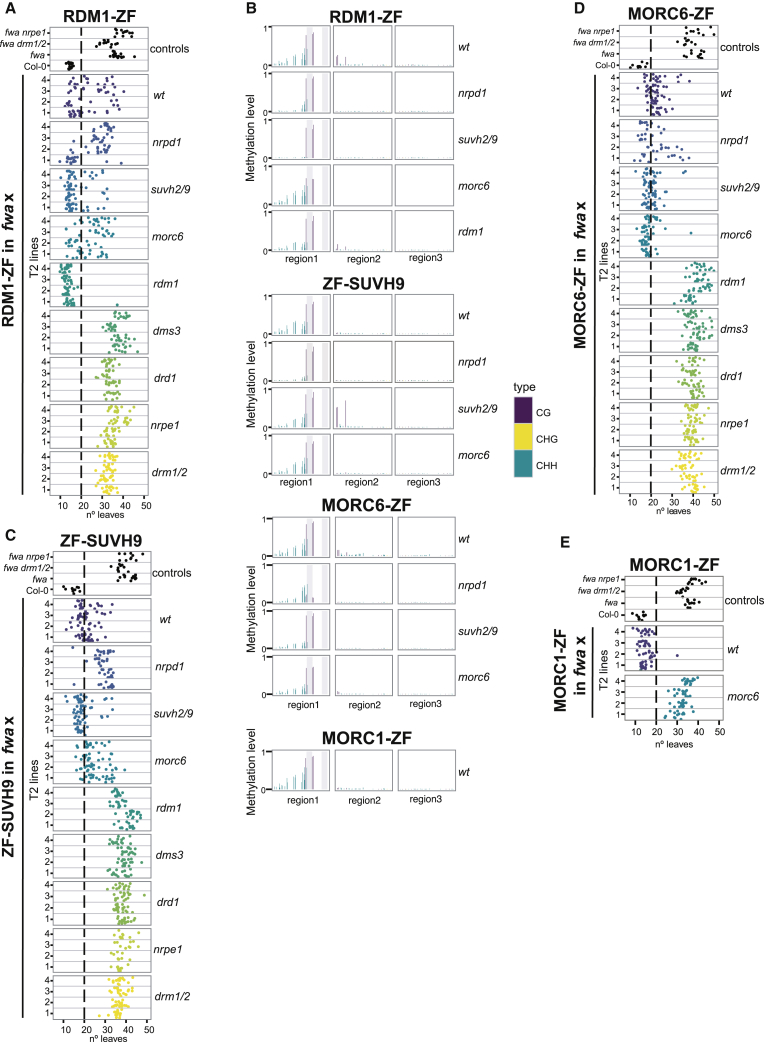
RDM1-, SUVH9-, and MORC-Mediated Targeted Methylation (A) Flowering time of control lines and four representative RDM1-ZF T2 lines in wild-type or different mutants that had been introgressed into the *fwa* background. (B) CG, CHG, and CHH DNA methylation over the *FWA* promoter measured by BS-PCR-seq in representative RDM1-ZF, ZF-SUVH9, MORC6-ZF, and MORC1-ZF T2 lines in wild-type or different mutants introgressed into *fwa*. Gray vertical lines indicate the ZF binding sites. (C) Flowering time of four representative ZF-SUVH9 T2 lines in different mutant backgrounds. (D) Flowering time of four representative MORC6-ZF T2 lines in different mutant backgrounds. (E) Flowering time of four representative MORC1-ZF T2 lines in different mutant backgrounds. See also Figure S3.

Combining ZF-RdDM fusions with RdDM mutations offered a unique approach to interrogate the hierarchy of action of RdDM proteins in *de novo* DNA methylation. Thus, we transformed ZF-RdDM fusion constructs into RdDM mutants that had been introgressed into the *fwa* background and scored flowering time of T1 populations (Figures S1, S2, and S3). We also confirmed flowering phenotypes in T2 plants descended from the four earliest T1 plants (Figures 1A, 1D, 1E, 2A, 3A, 3C, 3D, 3E, and S3E) and confirmed *FWA* methylation in representative T2 lines for all fusions that triggered early flowering (Figures 1B, 2B, 3B, and S3G). We also confirmed the lack of methylation in backgrounds where the representative fusions of the first arm (NRPD1-ZF), the second arm (DMS3-ZF), and ZF-DRMcd (Figures 1B, 2B, and S3G) did not induce early flowering.

### Ectopic Methylation Induced by RdDM Arm 1: siRNA Biogenesis

#### Targeting by NRPD1

NRPD1-ZF caused early flowering and *FWA* methylation in the *fwa* background (Figure 1A, 1B, and S1B). *SHH1* loss did not block targeted methylation and silencing, consistent with SHH1 acting upstream of Pol IV recruitment (Law et al., 2013) (Figure 1A, 1B, and S1B). Similar results were obtained in the *clsy1* mutant background (Figures 1A, 1B, and S1B), indicating that this chromatin remodeling protein is dispensable when Pol IV is artificially targeted to chromatin. However, NRPD1-ZF failed to trigger methylation in the *rdr2* mutant, consistent with previous observations that RDR2 is needed for P4-RNA production (Blevins et al., 2015, Li et al., 2015, Zhai et al., 2015) (Figures 1A, 1B, and S1B) and consistent with its proposed role downstream of Pol IV in the production of dsRNAs for siRNA biogenesis.

NRPD1-ZF induced *FWA* methylation in *dcl3*, as well as in *dcl2 dcl4*, indicating that different DCLs can process P4-RNAs into siRNAs for methylation targeting (Figures 1A, 1B, and S1B). This is consistent with the observation that DCL2 and DCL4 can produce 21- to 22-nt siRNAs in non-canonical Pol II-RDR6 RdDM (Cuerda-Gil and Slotkin, 2016). Moreover, NRPD1-ZF triggered *FWA* methylation in *dcl2 dcl3 dcl4* triple-mutant plants (Figure 1B). However, contrary to all other mutant backgrounds, we only observed early-flowering plants in the T2, not the T1 generation (Figures 1A and S1B), indicating that methylation targeting was less efficient compared to single or double *dcl* mutants. A similar observation was reported using VIGS to target methylation to *FWA* (Bond and Baulcombe, 2015).

To analyze siRNA biogenesis in different *DCL* mutant backgrounds containing NRPD1-ZF, we performed small RNA sequencing (sRNA-seq) in T2 lines. We did not detect *FWA* siRNAs in the unmethylated *fwa* epiallele, and NRPD1-ZF triggered the production of mostly 24-nt siRNAs and some 21-nt and 22-nt siRNAs (Figures 1C and S1C). As expected, *dcl3* mainly reduced 24-nt *FWA* siRNAs (Figures 1C and S1C). The *dcl3* and especially the *dcl2 dcl3 dcl4* mutants also accumulated longer sRNAs (up to 36 nt) (Figure S1D), likely corresponding to unprocessed P4-RNAs (Kuo et al., 2017), and likely accounting for some of the 21- to 24-nt signal remaining in these mutants (Figure 1C). Although we were unable to test the contribution of DCL1 due to lethality of *dcl1* null mutants, the biogenesis of the remaining sRNAs in *dcl2 dcl3 dcl4* is likely the consequence of DCL1 activity or due to DCL-independent activities (Kuo et al., 2017).

NRPD1-ZF triggered early flowering and methylation in *ago4* and *ago6 ago9* mutants (Figures 1A, 1B, and S1B), suggesting that these AGOs are functionally redundant in mediating *FWA* methylation. However, NRPD1-ZF did not trigger methylation in *ago4 ago6 ago9*, highlighting the importance of these three AGOs for targeted *de novo* methylation (Figures 1A, 1B, and S1B). Similarly, NRPD1-ZF failed to trigger methylation in the Pol V mutant *nrpe1* or in *drm1 drm2* double mutants (*DRM1* is a lowly expressed DRM2 homolog), consistent with a requirement for these RdDM components downstream of siRNA biogenesis (Figures 1A, 1B, and S1B). Lastly, a *CHROMOMETHYLASE 3* (*CMT3*) mutant did not block targeted *FWA* methylation (Figures 1A, 1B, and S1B).

Targeted methylation with ZF-SUVH9 (which recruits Pol V) was previously shown to be heritable after segregating away the ZF-SUVH9 transgene (Johnson et al., 2014). We found that methylation targeted by NRPD1 was also heritable, since plants that had segregated away the NRPD1-ZF transgene were early flowering and showed *FWA* methylation similar to plants that carried the transgene (Figures S1E and S1F).

#### Targeting by RDR2

RDR2-ZF induced DNA methylation and silencing of *FWA*, with a methylation pattern similar to that of NRPD1-ZF (Figures 1B, 1D, and S1G). However, RDR2-ZF failed to trigger early flowering in *nrpd1* (Figures 1D and S1G), consistent with the strong association of RDR2 with the Pol IV complex and its role in converting P4-RNAs into dsRNA. RDR2-ZF behaved similarly to NRPD1-ZF in all other tested mutant backgrounds, except for its ability to induce *FWA* silencing in the *rdr2* mutant, as predicted (Figures 1B, 1D, and S1G).

#### Targeting by SHH1

SHH1-ZF triggered *FWA* silencing and methylation (Figures 1B, 1E, and S1H). As expected for a Pol IV recruitment factor, SHH1-ZF could not induce *FWA* silencing in *nrpd1* or *rdr2* mutants or in *nrpe1* and *drm1 drm2* mutants (Figures 1E and S1H). Interestingly, SHH1 could induce *FWA* methylation in *clsy1*, suggesting that SHH1 can act independently of this factor (Figures 1B, 1E, and S1H). Contrary to NRPD1-ZF and RDR2-ZF, SHH1-ZF-targeted methylation was concentrated in a smaller region flanking the ZF binding region (Figure 1B). However, methylation was more extensive when SHH1-ZF was targeted in an *shh1* mutant (Figure 1B), which correlated with an enhanced frequency of early-flowering T1 plants in *shh1* (Figure S1H). This finding suggests that endogenous SHH1 competes with SHH1-ZF for Pol IV targeting.

### Ectopic Methylation Induced by RdDM Arm 2: Pol V Recruitment and Methylation Targeting

#### Targeting by DMS3

DMS3-ZF triggered the highest frequency of early flowering in T1 plants of any other factor (Figure S2A). DMS3 was efficient in targeting methylation in the *suvh2 suvh9* double mutant and in the *morc6* mutant, positioning DMS3 downstream of these components (Figures 2A, 2B, and S2A). DMS3-ZF activity was blocked in *nrpe1*, as well as in a mutant of another DDR component, *DRD1* (Figures 2A, 2B, and S2A), consistent with the role of DMS3 as a DDR complex component needed for Pol V recruitment (Zhong et al., 2012). However, it could target methylation (though less efficiently) in plants containing a mutation in the third DDR component, *RDM1* (Figures 2A, 2B, and S2A). One interpretation of this result is that RDM1 functions in the recruitment or stabilization of the DDR complex to chromatin, a function that can be replaced by artificially tethering DMS3 to chromatin.

Unexpectedly, DMS3 caused early flowering and methylation in the *nrpd1* mutant, suggesting that successful *de novo* methylation could be established in the absence of siRNAs (Figures 2A, 2B, and S2A). To confirm this hypothesis, we sequenced sRNAs in lines expressing DMS3-ZF in wild-type or *nrpd1* mutant backgrounds (Figures 2C and S2B). We observed high levels of 24-nt siRNAs and some 21-nt and 22-nt siRNAs over the ZF binding region in *fwa* plants expressing DMS3-ZF, but not in DMS3-ZF *nrpd1* mutant plants (Figures 2C and S2B). Furthermore, DMS3-ZF also targeted methylation in an *rdr1 rdr6* double mutant and an *rdr1 rdr2 rdr6* triple mutant, reinforcing the idea that DMS3 may induce methylation in the absence of siRNAs (Figures 2A, 2B, and S2A). While it seems unlikely, we cannot rule out, however, that trace levels of siRNAs from some unknown source are involved in the process.

As expected, DMS3-ZF failed to target methylation in *drm1 drm2* double mutant (Figure 2B), but surprisingly, a number of independent transgenic lines exhibited a mild early-flowering phenotype (Figures 2A and S2A), suggesting that DMS3-ZF could suppress *FWA* without inducing DNA methylation. Consistent with this hypothesis, RNA-seq of three independent early-flowering T2 lines showed a partial repression of *FWA* by DMS3-ZF in the *drm1 drm2* background (Figure S2C). DMS3-ZF was able to efficiently recruit Pol V at *FWA* in both wild-type or *drm1 drm2* mutant (Figure 2D), supporting a DNA-methylation-independent role of DDR in recruiting Pol V. Furthermore, the fact that DMS3-ZF plants showed early flowering in *drm1 drm2*, but not in *nrpe1*, suggests that Pol V recruited by DMS3-ZF in a *drm1 drm2* mutant might be interfering with Pol II transcription. Indeed, Pol II chromatin immunoprecipitation (ChIP)-seq showed lower Pol II occupancy in DMS3-ZF lines in the *drm1 drm2* background than in the *nrpe1* background (Figure S2D).

DMS3-ZF was able to target methylation in an *ago6 ago9* mutant (Figures 2A, 2B, and S2A). However, targeted methylation was greatly reduced in *ago4* and totally blocked in the *ago4 ago6 ago9* triple mutant (Figures 2A, 2B, and S2A), showing that an ARGONAUTE of the AGO4/6/9 clade is crucial for DMS3-dependent targeted methylation. This result, coupled with the fact that DMS3 appears to target methylation in an siRNA-independent manner, suggests that unloaded AGO protein may be sufficient to physically “bridge” Pol V and DRM2. This would be consistent with the known physical interactions between AGO4 and Pol V (El-Shami et al., 2007, Li et al., 2006) and between AGO4 and DRM2 (Zhong et al., 2014). Importantly, DMS3-ZF was still able to recruit Pol V in *ago4 ago6 ago9* (Figure 2D) and caused an intermediate early-flowering phenotype, consistent with the Pol V repressive effect on *FWA* expression also observed in the DMS3-ZF lines in *drm1 drm2*. Lastly, *cmt3* mutant did not block targeted methylation (Figures 2A, 2B, and S2A).

#### Targeting by RDM1

RDM1-ZF caused *FWA* methylation and early flowering, although with much lower efficiency than DMS3-ZF (Figures 3A and S3A). Consistent with the DMS3 results, RDM1 induced *FWA* methylation in *nrpd1*, *suvh2 suvh9*, and *morc6* mutants (Figures 3A, 3B, and S3A), further supporting the notion that Pol V recruitment through the DDR complex can be sufficient to initiate RdDM. Interestingly, RDM1-ZF was more efficient when transformed into an *rdm1* mutant (Figures 3A and S3A), suggesting that endogenous RDM1 might compete with RDM1-ZF’s ability to recruit or interact with the other DDR components. RDM1 was not able to cause early flowering in *drd1*, *dms3*, *nrpe1*, *and drm1 drm2* mutants (Figures 3A and S3A), indicating that RDM1 is unable to recruit Pol V in the absence of the other DDR complex components.

#### Targeting by SUVH9

ZF-SUVH9 targeted methylation in wild-type plants, as well as in *nrpd1* (with lower efficiency), but not in any of the DDR complex single mutants, *nrpe1*, or *drm1 drm2*, positioning SUVH9 upstream of DDR/Pol V (Figures 3B, 3C, and S3B). Although SUVH9 can interact with MORC6 (Jing et al., 2016, Liu et al., 2014), it was able to efficiently trigger methylation in a *morc6* mutant (Figures 3B, 3C, and S3B), indicating that SUVH9 can act independently of this factor.

#### Targeting by MORC6

MORC6-ZF triggered early flowering and induced *FWA* methylation in wild-type and *nrpd1* mutant backgrounds but could not trigger methylation in mutants of the DDR complex, *nrpe1* or *drm1 drm2* (Figures 3B, 3D, and S3C), suggesting that MORC6-ZF acts upstream of DDR to recruit Pol V activity.

Considering that both MORC6-ZF and ZF-SUVH9 act upstream of DDR/Pol V activity, we tested the ability of MORC6-ZF to target methylation in *suvh2 suvh9* and found that it did so efficiently (Figures 3B, 3D, and S3C). This result, together with the observation that ZF-SUVH9 can target methylation in the *morc6* mutant (Figures 3B, 3C, and S3B), positions these two fusion proteins in parallel pathways that utilize the DDR complex to recruit Pol V and establish DNA methylation. Indeed, Pol V ChIP-seq showed that both MORC6-ZF and ZF-SUVH9 were able to recruit Pol V in either a wild-type or *drm1 drm2* background (Figure 2D). However, both were somewhat less efficient at recruiting Pol V than DMS3-ZF, likely explaining why they did not cause *FWA* silencing in a *drm1 drm2* background (Figures 3C, 3D, S3B, and S3C).

Consistent with MORC1's ability to form heterodimers with MORC6 (Moissiard et al., 2014), MORC1-ZF was also able to induce *FWA* methylation and silencing, and this ability was abolished in the absence of MORC6 (Figures 3B, 3E, and S3D).

### Ectopic Methylation by the DRM Catalytic Domain

We found that ZF fused to the tobacco DRMcd that had been previously crystallized (Zhong et al., 2014) induced *FWA* silencing and methylation (Figures S3E–S3G). The *cmt3* mutant did not affect ZF-DRMcd activity. Unexpectedly, however, ZF-DRMcd activity was greatly reduced in *nrpd1* and completely blocked by *nrpe1* or *drm1 drm2* mutations (Figures S3E–S3G), suggesting that an active RdDM pathway is needed to perpetuate or amplify the methylation seeded by the DRM catalytic domain.

### DMS3-ZF Triggers DNA Methylation at Additional Sites

Zinc fingers are rarely highly specific in their binding, and we therefore sought to take advantage of “off-target” binding by ZF to study DNA-methylation targeting at additional sites in the genome. We focused our initial analysis on the highly efficient RdDM fusion, DMS3-ZF. The DNA-methylation landscape of the *fwa-4* epimutant is chimeric, since it was generated by crossing wild-type Col-0 with *met1* mutant plants. Because this might complicate analysis of targeted DNA methylation, we re-transformed DMS3-ZF, as well as a control construct containing the ZF alone, into wild-type Col-0 plants and performed ChIP-seq to identify ZF off-target sites.

**Figure 4:**
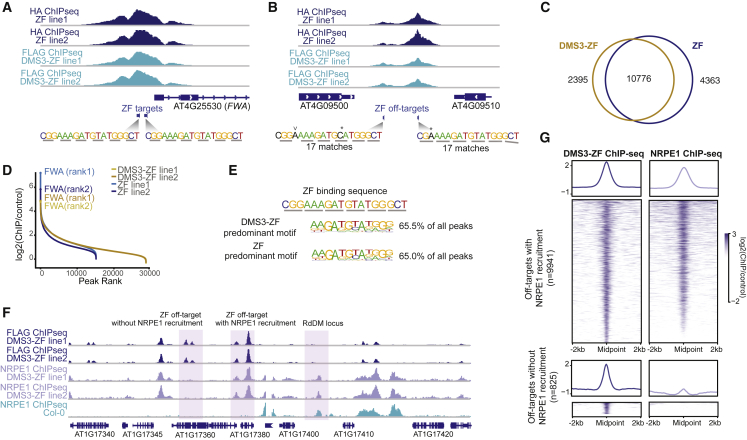
DMS3-ZF Efficiently Recruits Pol V to Thousands of Loci (A) Screenshot of ZF ChIP-seq over *FWA* in two independent DMS3-ZF and ZF control lines. ZF binding sites and sequence are depicted. (B) Screenshot of ZF ChIP-seq over two off-target sites in DMS3-ZF and ZF control lines. Similar sequences to ZF binding sequence (17-bp match) are depicted. Asterisk indicates nucleotide substitution. “v” indicates nucleotide insertion. (C) Overlap between ChIP-seq peaks in DMS3-ZF and ZF lines using 2-fold change compared to control ChIP-seq in Col-0 as a cutoff. (D) Inflection curves of ChIP-seq peaks for the two DMS3-ZF and ZF lines are shown. Peak intensity compared to control (FLAG and hemagglutinin [HA] ChIP-seq in Col-0) is shown on the y axis and peak rank based on peak intensity is shown on the x axis. (E) Predominant motif identified by *de novo* motif analysis for DMS3-ZF and ZF peaks. (F) Screenshot of DMS3-ZF and NRPE1 ChIP-seq in two independent DMS3-ZF lines and NRPE1 ChIP-seq in a Col-0 control. (G) Metaplot and heatmap of DMS3-ZF and NRPE1 ChIP-seq signals in DMS3-ZF off-target sites with (upper panels) or without (lower panels) NRPE1 recruitment. See also Figure S4.

**Figure S4:**
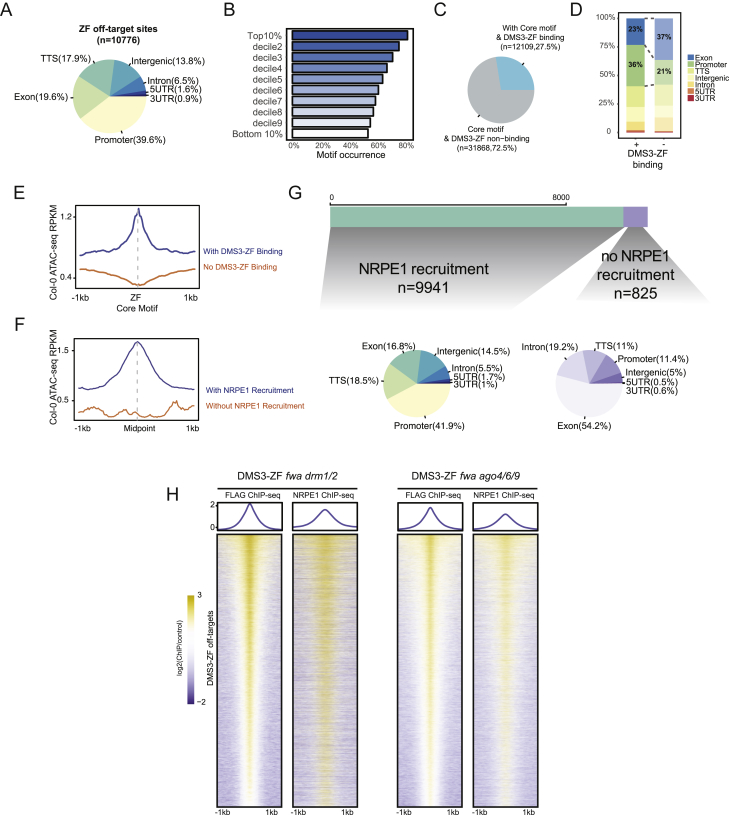
DMS3-ZF Efficiently Recruits Pol V to Thousands of Loci, Related Figure 4 (A) Pie chart of the genomic distribution of off-target sites in common between DMS3-ZF and ZF. (B) Bar plot showing the motif occurrence percentage over different deciles of DMS3-ZF peaks based on their enrichment. (C) Pie chart showing the percentage of DMS3-ZF binding sites over ZF core motif-containing sites. (D) Genomic distribution of core motif-containing sites with or without DMS3-ZF binding. (E) Metaplot of Col-0 ATAC-seq signals over core motif-containing sites with or without DMS3-ZF binding. (F) Metaplot of Col-0 ATAC-seq signals over ZF off-target sites with or without NRPE1 recruitment in DMS3-ZF. (G) Genomic distribution of ZF off-target sites with (left) or without (right) NRPE1 recruitment in DMS3-ZF. (H) Heatmap and metaplot showing FLAG and NRPE1 ChIP-seq signal in DMS3-ZF lines in *fwa drm1/2* (left panel) and *fwa ago4/6/9* (right panel) over DMS3-ZF off-targets.

DMS3-ZF and ZF had similar binding patterns and were found at thousands of loci (Figures 4A–4C), showing a preference for promoter regions (Figure S4A). When we ranked the ChIP-seq peak signals across the genome, the *FWA* peak ranked first or second in both DMS3-ZF and ZF control lines (Figure 4D); however, there were also many additional strong peaks. The DMS3-ZF ChIP-seq peak intensities strongly correlated with the presence of the ZF binding sequence (Figure S4B). A *de novo cis*-motif analysis identified a core motif sequence corresponding to the inner ZF repeats of ZF as the most overrepresented (Figure 4E), suggesting that the external two ZF repeats do not play a major role in the specificity of ZF binding to chromatin. Despite the fact that the ZF inner core motif was highly abundant in the genome, only 27.5% of the loci containing this motif were occupied by ZF fusions (Figure S4C). When we analyzed the genome-wide ZF motif distribution with respect to the presence or absence of ZF binding, we observed that ZF fusions tended to bind the motif when present in promoters and tended to be excluded from motifs present in exons (Figure S4D). These differences might be due to differences in chromatin accessibility. Indeed, among the loci that contain a ZF binding motif, those bound by DMS3-ZF showed a more open chromatin structure as measured by ATAC-seq (Lu et al., 2017) (Figure S4E), suggesting that chromatin accessibility could be a major determinant for ZF binding to its targets.

To test the efficiency of DMS3-ZF in recruiting Pol V at different loci, we performed ChIP-seq of the Pol V catalytic subunit NRPE1. Strikingly, over 90% of DMS3-ZF binding sites gained a Pol V peak (Figures 4F and 4G). Consistent with the ZF binding profile, DMS3-dependent Pol V recruitment was more efficient over open chromatin regions like promoters and tended to be excluded from exons (Figures S4F and S4G). We also explored the ability of DMS3-ZF to recruit Pol V in the absence of targeted methylation or AGO proteins by performing Pol V ChIP-seq in *drm1 drm2* or *ago4 ago6 ago9* backgrounds. Like at *FWA*, these mutations did not reduce the ability of DMS3-ZF to recruit Pol V throughout the genome (Figure S4H).

**Figure 5:**
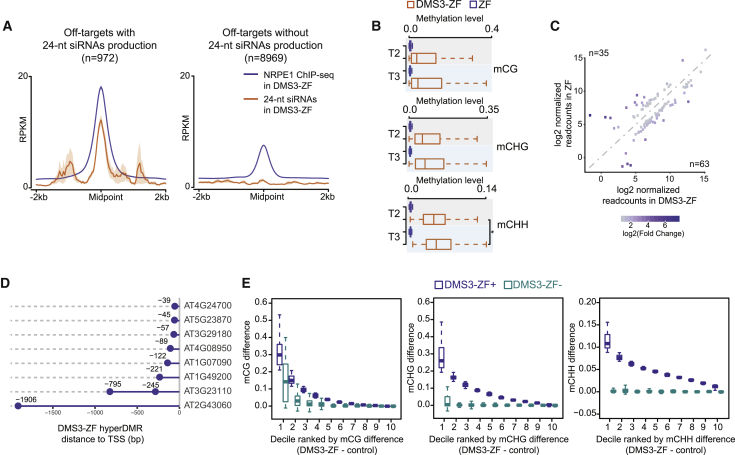
DMS3-ZF Targets Methylation to Hundreds of Loci (A) Metaplot of NRPE1 ChIP-seq and 24-nt siRNAs in DMS3-ZF over off-target sites with NRPE1 and with (left) or without (right) 24-nt siRNAs production. Shaded area around each curve represents standard errors. y axis represents reads per kilobase million (RPKM). (B) CG, CHG, and CHH methylation levels in T2 and T3 DMS3-ZF and ZF over 24-nt siRNA-producing off-target sites with hyperDMRs. ^∗^p < 0.05 (Welch two-sample t test). (C) Log2-normalized-readcount scatterplot of differentially expressed genes between DMS3-ZF and ZF T3 lines. (D) Distance of hypermethylated off-targets in DMS3-ZF to the nearest TSS of downregulated genes in DMS3-ZF compared to ZF. (E) Boxplots showing the gain of CG, CHG, and CHH methylation (y axis) in DMS3-ZF T2 plants that contain (+) or have segregated the transgene away (−). x axis represents DNA methylation levels (DMS3-ZF − control) divided into deciles ordered from higher (1) to lower (10). Control represents the average methylation of different ZF and Col-0 lines (see STAR Methods). See also Figure S5.

**Figure S5:**
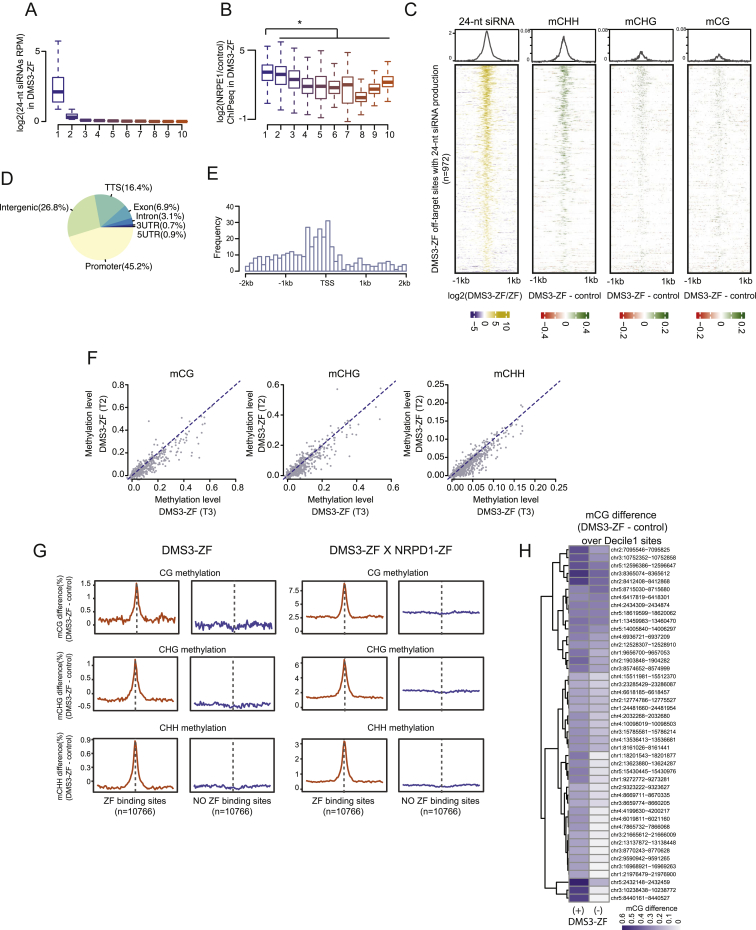
DMS3-ZF Targets Methylation to Hundreds of Loci, Related Figure 5 (A) Boxplot of 24-nt siRNAs levels for deciles ordered by 24-nt siRNAs levels over DMS3-ZF off-target sites. (B) Boxplot of NRPE1 levels in DMS3-ZF over different 24-nt siRNAs deciles as shown in (A). ^∗^p < 0.05 (Welch Two Sample t test). (C) Heatmap showing 24-nt siRNAs production (DMS3-ZF over ZF control) and CHH, CHG and CG methylation difference (DMS3-ZF minus control) over DMS3-ZF off-targets that produce siRNAs. Control represents the average methylation of different ZF and Col-0 lines (see methods). (D) Genomic distribution of off-target sites with NRPE1 recruitment, 24-nt siRNA production, and hyperDMR in DMS3-ZF. (E) Frequency of off-target sites with NRPE1 recruitment, 24-nt siRNA production, and hyperDMR in DMS3-ZF within 2kb upstream and downstream of annotated genes. (F) Scatterplot of CG, CHG and CHH methylation in DMS3-ZF T2 and T3 lines. Dashed lines provide visual assistance. (G) Metaplot of CG, CHG and CHH methylation difference over ZF binding sites (n = 10766) and same number of random shuffled sites without ZF binding in DMS3-ZF and DMS3-ZF X NRPD1-ZF. Control represents the average methylation of different ZF and Col-0 lines (see methods). (H) Heatmap showing CG methylation increase in regions that belong to the decile 1 from Figure 5E in DMS3-ZF that contain (+) or have segregated the transgene away (-). Coordinates for each loci are indicated.

The promiscuous nature of DMS3-ZF binding and its high efficiency of Pol V recruitment provided a unique opportunity to study the ability of Pol V to target methylation to thousands of loci. We first examined siRNA production over DMS3-ZF binding regions that recruited Pol V (n = 9,941; Figure 4G). Compared to the high efficiency in recruiting Pol V, only 9.8% (n = 972) of the Pol-V-containing DMS3-ZF off-targets showed *de novo* accumulation of 24-nt siRNAs (Figure 5A). In addition, the loci in which siRNA production was stimulated by DMS3-ZF corresponded to those with the highest Pol V recruitment (Figures 5A, S5A, and S5B). This suggests that high levels of Pol V recruitment are needed to engage the full RdDM pathway and stimulate siRNA production.

To study targeted methylation at these sites, we analyzed whole-genome DNA methylation of T2 and T3 plants expressing DMS3-ZF and ZF, as well as Col-0 controls. Of the DMS3-ZF loci producing siRNAs, most showed some hypermethylation (Figure S5C), and 46% (n = 451) were called as differentially hypermethylated regions (hyperDMRs) with stringent criteria. Consistent with the genomic distribution of ZF and its correlation with open chromatin (Figures S4A and S4E), this set of siRNA-producing, hyperDMR loci was highly enriched over promoters and intergenic regions (Figure S5D), and the number of methylated loci increased with proximity to transcriptional start sites (TSSs) (Figure S5E). The methylation at these sites also increased somewhat from the T2 to the T3 generation (Figures 5B and S5F). Methylation targeting was specific for ZF-bound loci because we observed a peak of CG, CHG, and CHH methylation over a metaplot of the 10,766 ZF-bound sites but no methylation targeting over a random set of 10,766 non-ZF-bound sites (Figure S5G, left panels).

In order to study effects on gene expression, we performed RNA-seq in DMS3-ZF and ZF plants. 63 genes were upregulated and 35 were downregulated in DMS3-ZF plants compared to ZF plants (Figure 5C). Of the 35 downregulated genes, eight showed overlap with the hyperDMR regions bound by DMS3-ZF. Consistent with the observation that DNA methylation has a stronger impact on gene expression when it is close to the TSS (Zhong et al., 2012), seven of these eight downregulated genes had hypermethylation within 250 bp upstream of the TSS (Figure 5D).

We also examined the heritability of targeted methylation by analyzing the hypermethylated regions in T2 plants that contained the transgene (DMS3-ZF+) or that had segregated it away (DMS3-ZF−). We divided these methylated regions into three sequence contexts—CG, CHG, and CHH—and ranked them in deciles based on the methylation difference between DMS3-ZF and control plants. We found that regions with higher CG methylation showed much higher heritability in the absence of the transgene compared to CHG- or CHH-methylation-enriched regions (Figure 5E). We also calculated the difference in methylation levels between DMS3-ZF+ and DMS3-ZF− plants at the hypermethylated regions corresponding to the first decile defined in Figure 5E, where targeted CG methylation and heritability were the highest. We found that the methylation at 27 out 45 of these sites (60%) was heritable if we defined a heritable site as showing an increase of 10% CG methylation between DMS3-ZF− and control plants (Figure S5H). These results indicate that higher levels of targeted CG methylation are needed in order to have successful maintenance of ectopic DNA methylation in the absence of the trigger construct.

### siRNA-Independent AGO Recruitment and Targeted Methylation

**Figure 6:**
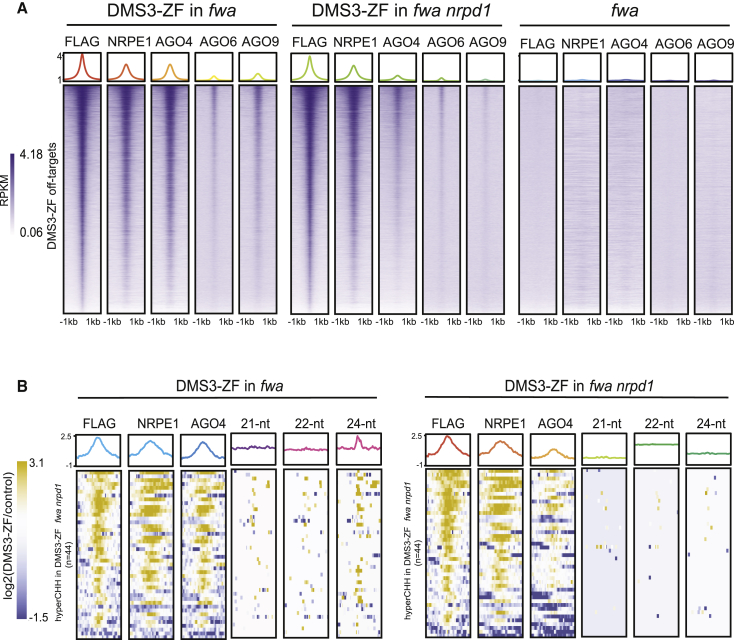
AGO Recruitment and siRNA-Independent Targeted Methylation (A) Heatmap and metaplot depicting ChIP-seq signals of FLAG (DMS3-ZF), NRPE1, AGO4, AGO6, and AGO9 over DMS3-ZF off-targets in DMS3-ZF lines in *fwa* (left panel), *fwa nrpd1* (center panel), and untransformed *fwa* (right panel). (B) Heatmap and metaplot depicting ChIP-seq signals of FLAG (DMS3-ZF), NRPE1 and AGO4, and sRNA-seq signal for 21-nt, 22-nt, and 24-nt siRNAs in DMS3-ZF in *fwa* (left panel) and *fwa nrpd1* (right panel) backgrounds over hypermethylated regions in DMS3-ZF in *fwa nrpd1* (n = 44). See also Figure S6.

**Figure S6:**
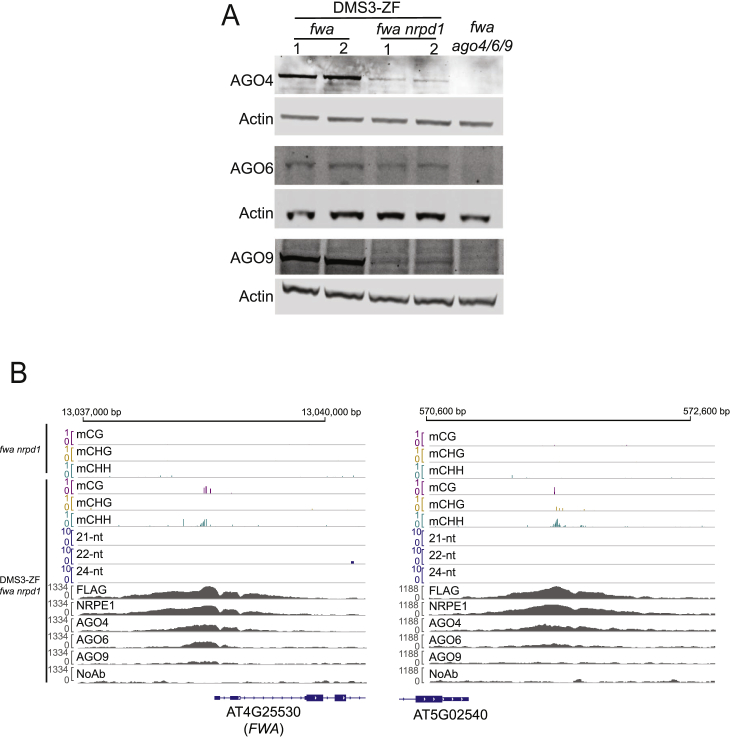
AGO Recruitment and siRNA-Independent Targeted Methylation, Related Figure 6 (A) Western Blot depicting AGO4, AGO6, AGO9 and Actin in two independent DMS3-ZF lines in *fwa* and *fwa nrpd1* backgrounds and untransformed *fwa ago4 ago6 ago9* (*fwa ago4/6/9)* control. (B) Screenshots showing two DMS3-ZF hypermethylated regions in *fwa nrpd1* background. Y axis for CG, CHG, and CHH tracks represent methylation level. Y axis for 21-nt, 22-nt and 24-nt siRNAs and FLAG, NRPE1, AGO4, AGO6, AGO9 and no antibody (NoAb) control ChIP tracks represent RPKM.

DMS3-ZF can target *FWA* methylation in the absence of Pol IV but still requires AGO4, AGO6, and/or AGO9 (Figure 2B), suggesting that DMS3-ZF may still recruit these AGOs to chromatin in the absence of siRNAs. To test this, we performed AGO4, AGO6, and AGO9 ChIP-seq in DMS3-ZF lines in wild-type and *nrpd1* backgrounds. We observed a very high overlap between the different AGOs and DMS3-ZF off-target sites (Figure 6A). Strikingly, the AGOs were also still bound to these regions in an *nrpd1* mutant, although the signal strength was reduced (Figure 6A). This reduction is consistent with the previously reported reduced abundance of the AGO4, AGO6, and AGO9 proteins as measured by western blots in *nrpd1* (Figure S6A) (Havecker et al., 2010, Li et al., 2006).

The number of DMS3-ZF-dependent hypermethylated off-targets showed a dramatic reduction in the *nrpd1* mutant (only 44 sites remaining), indicating that Pol IV-dependent siRNAs are required for efficient targeted methylation at most sites but also that targeted methylation in a Pol IV mutant is not a unique feature of *FWA* (Figure S6B). At these 44 sites, there was a clear 24-nt siRNA accumulation in wild-type, but not in the *nrpd1* background (Figure 6B). This is consistent with the *FWA* results (Figures 2C and S2B) and supports a model where targeted methylation can happen in the absence of siRNAs, although with much lower efficiency.

### Co-targeting of DMS3 and NRPD1 Enhances Ectopic RdDM

**Figure 7:**
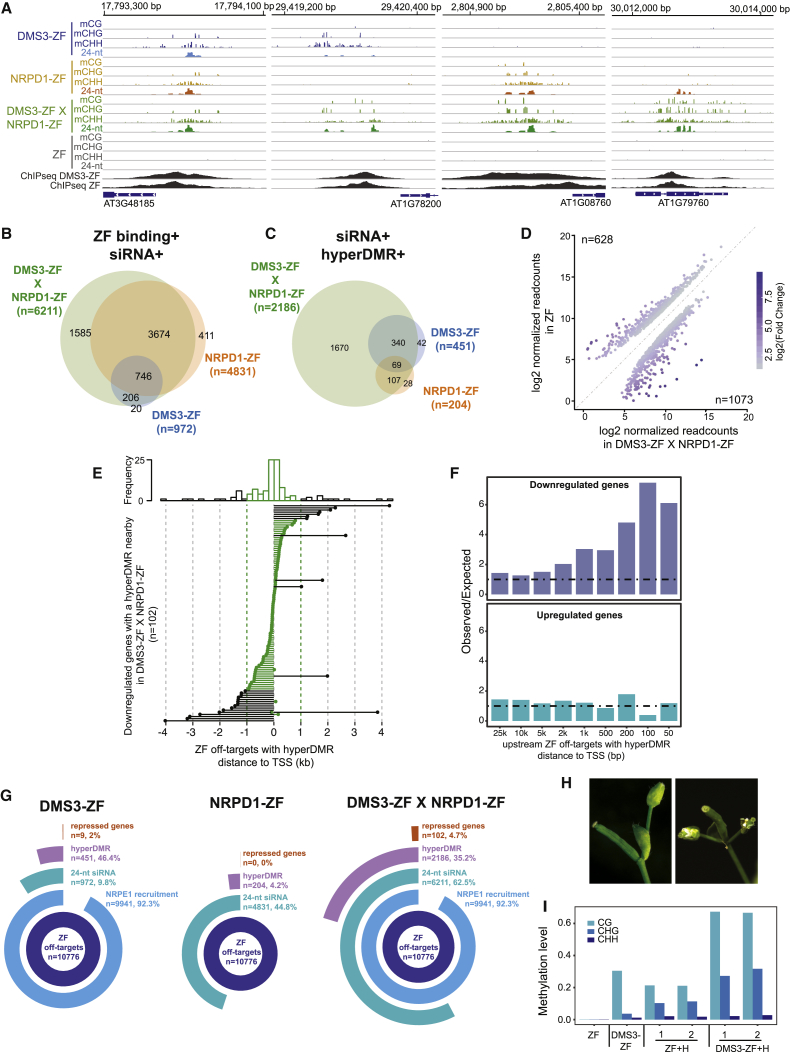
Co-targeting Pol IV and Pol V Promotes Efficient Ectopic DNA Methylation (A) Screenshots of CG, CHG, and CHH methylation and 24-nt siRNA levels in DMS3-ZF, NRPD1-ZF, DMS3-ZF X NRPD1-ZF, and ZF, as well as DMS3-ZF and ZF ChIP-seq over representative hypermethylated regions in DMS3-ZF, NRPD1-ZF, DMS3-ZF X NRPD1-ZF (left), DMS3-ZF and DMS3-ZF X NRPD1-ZF (middle left), NRPD1-ZF and DMS3-ZF X NRPD1-ZF (middle right), and DMS3-ZF X NRPD1-ZF only (right). (B) Venn diagram of 24-nt siRNAs-producing off-target sites in DMS3-ZF, NRPD1-ZF, and DMS3-ZF X NRPD1-ZF. (C) Venn diagram of 24-nt siRNAs-producing off-target sites with hyperDMR in DMS3-ZF, NRPD1-ZF, and DMS3-ZF X NRPD1-ZF. (D) Log2-normalized-readcounts scatterplot of differentially expressed genes in DMS3-ZF X NRPD1-ZF compared to ZF. (E) Histogram (upper panel) and lollipop plot (lower panel) showing the enrichment and distance of DMS3-ZF X NRPD1-ZF hyperDMRs to the nearby downregulated genes in DMS3-ZF X NRPD1-ZF compared to ZF. (F) Observed-over-expected ratio for down- and upregulated genes in DMS3-ZF X NRPD1-ZF proximal to ZF off-targets with hyperDMR. (G) Multilevel pie chart of the number of ZF off-target sites, NRPE1 recruited sites, 24-nt siRNA-producing sites, hyperDMRs, and repressed genes in DMS3-ZF (left), NRPD1-ZF (middle), and DMS3-ZF X NRPD1-ZF (right). (H) Two representative DMS3-ZF+H plants showing ectopic flowers characteristic of *ap1* mutants. (I) CG, CHG, and CHH methylation levels over the *AP1* promoter measured by BS-PCR-seq in representative ZF and DMS3-ZF plants and one representative plant from two independent T2 lines of ZF+H and DMS3-ZF+H.

Because only a small proportion of Pol V-containing DMS3-ZF-bound sites displayed siRNAs and DNA methylation, we hypothesized that co-targeting of Pol IV (via NRPD1) and Pol V (via DMS3) might stimulate full RdDM activity and therefore increase the number of additional ZF targets that become methylated. We first analyzed genome-wide DNA methylation and siRNA production in T1 plants expressing NRPD1-ZF (Figure 7A). Roughly 45% (4,831) of the 10,776 ZF-bound loci produced 24-nt siRNAs (Figures 7B and 7G). However, only 4.2% (n = 204) of these siRNA-producing sites became methylated, representing an even lower efficiency for ectopic methylation than DMS3-ZF (Figures 7C and 7G). Moreover, we observed only minor changes in gene expression in NRPD1-ZF plants compared to ZF control lines, none of which overlapped with genes with hypermethylated regions (Figure 7G). This suggests that recruitment of the siRNA biogenesis machinery alone is not sufficient to target methylation at most loci.

**Figure S7:**
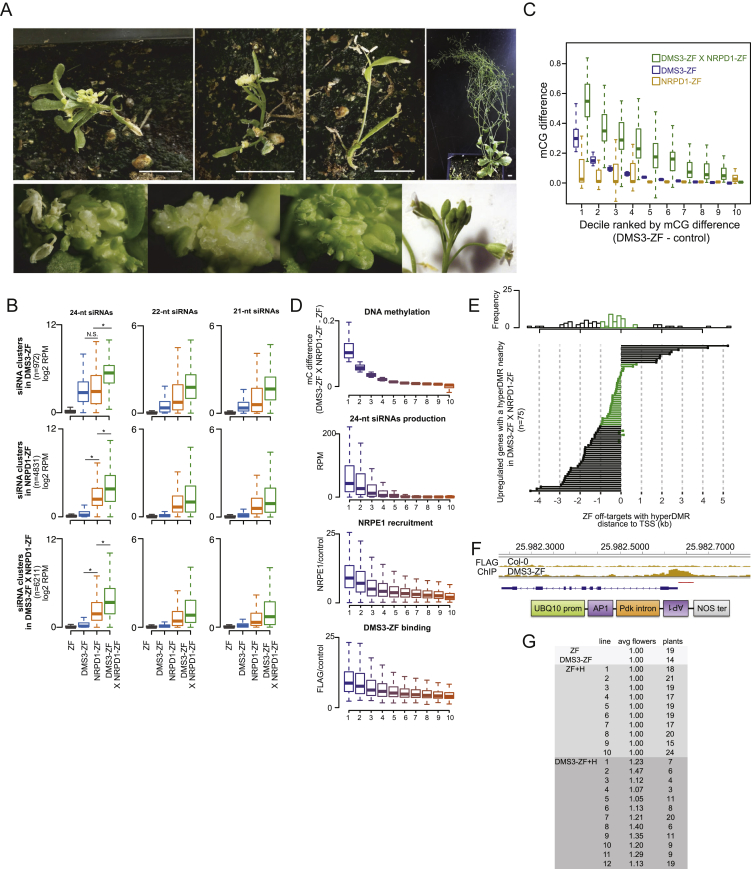
Co-targeting Pol IV and Pol V Promotes Efficient Ectopic DNA Methylation, Related Figure 7 (A) Abnormal phenotypes of DMS3-ZF X NRPD1-ZF plants. Upper left, upper-middle left and upper-middle right panels show 3 different DMS3-ZF X NRPD1-ZF mutant plants. Upper right panel shows a non mutant plant. White bar in the lower corner of each image = 1cm. Lower left, lower-middle left and lower-middle right panels show inflorescences of 3 different DMS3-ZF X NRPD1-ZF mutant plants. Lower right panel shows the inflorescence of a non mutant plant. (B) Boxplots of 24-nt, 22-nt and 21-nt siRNA levels in ZF, DMS3-ZF, NRPD1-ZF and DMS3-ZF X NRPD1-ZF over off-target sites producing 24-nt siRNAs in DMS3-ZF (upper), NRPD1-ZF (middle), and DMS3-ZF X NRPD1-ZF (lower). ^∗^p < 0.05 (Welch Two Sample t test). N.S. mean not significant. (C) Boxplot depicting CG DNA methylation difference in NRPD1-ZF, DMS3-ZF and DMS3-ZF X NRPD1-ZF lines. x axis represents DNA methylation levels (DMS3-ZF - control) divided in deciles ordered from higher (1) to lower (10). Control represents the average methylation of different ZF and Col-0 lines (see methods). (D) Boxplot of 10 deciles ordered by total methylation (CG/CHG/CHH combined) difference between DMS3-ZF X NRPD1-ZF and ZF over ZF off-target sites (n = 10766) (upper panel), normalized 24-nt siRNA levels (Reads Per Million, RPM) in DMS3-ZF X NRPD1-ZF over the 10 methylation difference deciles (upper middle panel), normalized NRPE1 abundance in DMS3-ZF (lower middle panel), and normalized DMS3-ZF binding in DMS3-ZF (lower panel). (E) Histogram (upper panel) and lollipop plot (lower panel) showing the enrichment and distance of DMS3-ZF X NRPD1-ZF hyperDMRs to the nearby upregulated genes in DMS3-ZF X NRPD1-ZF compared to ZF. (F) Screenshot depicting the ZF ChIP signal in DMS3-ZF and Col-0 control line over *AP1* (upper panel). The region targeted with the hairpin RNA construct is underlined in red. A representation of the hairpin RNA construct is shown (lower panel). (G) Table showing the average number of flowers per pedicel/peduncule present in the first 5 determinate structures in the main inflorescence (avg flowers). Data for ZF and DMS3-ZF controls, as well as different ZF+H and DMS3-ZF+H T2 lines is shown. Number of plants counted is shown in the right column (plants).

To study the possible synergistic effect of co-targeting Pol IV and Pol V, we supertransformed NRPD1-ZF into DMS3-ZF or ZF control lines. While ZF control lines expressing NRPD1-ZF did not show phenotypic changes compared to wild-type plants, lines expressing both DMS3-ZF and NRPD1-ZF showed a plethora of developmental defects, such as abnormal leaf, inflorescence, and floral patterning (Figure S7A) and complete infertility. sRNA-seq analysis showed an increase in the number of loci-producing siRNAs (Figure 7B), as well as an increase in the 21-nt, 22-nt, and 24-nt siRNAs levels compared to DMS3-ZF or NRPD1-ZF fusions alone (Figure S7B). Strikingly, 2,186 siRNA-producing sites were hypermethylated in the supertransformants, including almost all hypermethylated regions detected in either DMS3 or NRPD1 ZF lines (Figure 7C), which shows that co-targeting Pol IV and Pol V dramatically enhances the efficiency of ectopic, site-specific methylation. This synergy was also clear when we divided the hypermethylated regions into deciles ranked by methylation difference between the ZF fusions and control (Figure S7C). The methylation was also specific to the ZF-bound sites (Figure S5G, right panels). To gain additional insight into the contributions of ZF binding strength, Pol V recruitment levels, and siRNA abundance, we correlated their levels at each site with the levels of hypermethylation. This analysis showed that all three factors strongly correlated with the level of targeted methylation (Figure S7D).

We performed RNA-seq to identify genes whose expression might be affected by hypermethylation in plants expressing both DMS3-ZF and NRPD1-ZF. We found 628 downregulated and 1,073 upregulated genes in these plants compared to ZF controls (Figure 7D). 102 downregulated genes overlapped with hypermethylated regions, most of which were located within 1-kb regions proximal to the TSS (Figure 7E). In contrast, hypermethylated regions located close to upregulated genes were less abundant and were located more distant to TSSs (Figure S7E). We also adapted a published algorithm (Pastor et al., 2018) to determine if there is a correlation between the distance to the TSS of hyperDMR regions and repression or activation of gene expression. We observed that genes with hypermethylated regions within 200 bp upstream of their TSSs tended to be repressed, while no enrichment was observed for upregulated genes (Figure 7F). These results show that co-targeting of Pol IV and Pol V dramatically enhanced the number of misregulated genes associated with targeted DNA methylation (summarized in Figure 7G) and further underscores that proximity to TSSs is a factor to consider when targeting DNA methylation to repress gene expression.

We sought to exploit the enhanced targeted methylation achieved by combining Pol V and siRNAs to silence the floral master regulator *APETALA1* (*AP1*) (Irish and Sussex, 1990). DMS3-ZF shows a ChIP-seq peak in the *AP1* promoter region (Figure S7F). We created a hairpin construct to produce siRNAs directed against the *AP1* promoter and transformed it into DMS3-ZF (DMS3-ZF+H) or ZF (ZF+H) control plants (Figure S7F). All DMS3-ZF+H T2 lines exhibited a weak *ap1* mutant phenotype, with ectopic flowers developing in the axils of sepals (Figures 7H and S7G), while none of the ZF, DMS3-ZF, or ZF+H control lines did (Figure S7G). Consistent with the *ap1* phenotype, DMS3-ZF+H lines showed enhanced DNA methylation compared to DMS3-ZF or ZF+H control lines (Figure 7I). These results suggest that co-targeting siRNAs and Pol V can be an effective approach to achieve robust DNA methylation and gene silencing.

## Discussion

Our synthetic biology approach of combining gain-of-function ZF-RdDM fusions together with loss-of-function mutations allowed us to determine the hierarchy of action of a number of RdDM components, and also to identify RdDM factors that are most effective in targeting ectopic methylation and silencing. Of the 9 factors that could successfully target *FWA* methylation, the DDR component DMS3 was the most effective, and it could robustly recruit Pol V, AGO4, AGO6, and AGO9 and induce *FWA* DNA methylation and silencing even in the *nrpd1* mutant that eliminates siRNA biogenesis. However, DMS3-ZF targeted methylation was blocked in the *ago4 ago6 ago9* triple mutant. These results highlight an essential role of ARGONAUTE proteins in methylation targeting and suggest that unloaded AGO, or AGO loaded with non-complementary RNAs, can associate with Pol V and recruit DNA methyltransferase activity, thus triggering siRNA-independent DNA methylation. Our results also showed that tethering the Pol IV subunit NRPD1 to *FWA* was effective in promoting methylation and silencing, but it could not do so in the Pol V mutant *nrpe1*, highlighting an essential role for Pol V activity in *de novo* methylation targeting.

We also found that a MORC6 fusion was effective in recruiting Pol V, DNA methylation, and silencing, which was unexpected given that MORC6 mainly acts downstream of DNA methylation and does not have a typical RdDM phenotype (Harris et al., 2016, Matzke et al., 2015, Moissiard et al., 2012). Our interpretation of these results is that MORC6 may normally use its interaction with RdDM machinery as a mechanism for its own recruitment to facilitate its primary role in silencing that takes place downstream of DNA methylation. Artificial ZF tethering of MORC6 to chromatin likely reverses the normal situation and allows it to recruit the RdDM machinery *de novo*.

Our genome-wide analysis showed that DMS3-ZF was highly efficient at recruiting Pol V, as well as AGO4, AGO6, and AGO9, to thousands of off-target ZF sites. However, only a small fraction of these sites produced siRNAs and became methylated (Figure 7G). This indicates that, in contrast to the *FWA* locus, Pol V and AGO4/AGO6/AGO9 recruitment is not sufficient to recruit the entire RdDM pathway and target methylation at most loci. On the other hand, NRPD1-ZF was efficient at recruiting siRNA production to thousands of loci, but the number of these sites gaining methylation was even smaller than in DMS3-ZF plants (Figure 7G), indicating that recruitment of siRNA production alone is also not sufficient to target methylation at most loci. However, the co-targeting of Pol IV and Pol V activities by combining NRPD1-ZF and DMS3-ZF fusions synergistically enhanced the efficiency and resulted in the methylation of thousands of loci. This suggests that future strategies for efficient RdDM targeting should involve a combination of recruiting siRNA biogenesis and Pol V activity.

In summary, this work provides a theoretical framework for the design of efficient DNA-methylation targeting in plants. The factors identified in this work could be used with other programmable DNA-binding targeting platforms, such as CRISPR systems, to improve locus specificity and ease of multiplexed targeting.

## STAR★Methods

### Key Resources Table

**Table:** 

REAGENT or RESOURCE	SOURCE	IDENTIFIER
**Antibodies**		

Anti-FLAG	Sigma	Cat# F1804; RRID:AB_262044
Anti-HA	Roche	Cat# 11867423001; RRID:AB_2314622
Anti-Actin	Millipore	Cat# MAB1501; RRID:AB_2223041
Anti-RNA Polymerase II phospho S5	Abcam	Cat# Ab5131; RRID:AB_449369
Anti-NRPE1	Liu et al., 2018	N/A
Anti-AGO4	Dr Olivier Voinnet	N/A
Anti-AGO6	Dr Olivier Voinnet	N/A
Anti-AGO9	Dr Olivier Voinnet	N/A
Anti-mouse IgG 800	LI-COR	Cat# 925-32210
Anti-rabbit IgG 800	LI-COR	Cat# 925-32211

**Bacterial and Virus Strains**		

*Agrobacterium tumefaciens* AGL0	N/A	N/A
*Escherichia coli* DH10B	NEB	Cat# C3019L

**Chemicals, Peptides, and Recombinant Proteins**		

Complete EDTA-free Protease Inhibitor	Roche	Cat# 11836170001
Hygromycin B	Invitrogen	Cat# 10687010
Glufosinate ammonium	Goldbio	Cat# P-165-250
PMSF	SIGMA	Cat# P7626
MG132	TOCRIS	Cat# 1748
Pepstatin A	Fisher Scientific	Cat# BP26715
Protein A Dynabeads	Invitrogen	Cat# 10002D
Protein G Dynabeads	Invitrogen	Cat# 10004D
GlycoBlue	Invitrogen	Cat# AM9515

**Critical Commercial Assays**		

Direct-zol RNA kit	Zymo Research	Cat# R2050
Ovation Ultralow V2 kit	NuGEN	Cat# 0344NB-A01
Ovation Ultralow Methyl-Seq	NuGEN	Cat# 0335-32
TruSeq Nano DNA Library Prep kit for Neoprep	Illumina	Cat# NP-101-1001
TruSeq Stranded mRNA Library Prep kit for Neoprep	Illumina	Cat# NP-202-1001
TruSeq Stranded mRNA Library Prep kit	Illumina	Cat# 20020594
Kapa Hyper Prep kit	KAPA Biosystems	Cat# KK8502
Pfu Turbo Cx	Agilent	Cat# 600412
EpiTect Bisulfite Kit	QIAGEN	Cat# 59104
EZ DNA methylation-lighting kit	ZYMO research	Cat# D5030
TruSeq Small RNA Library Prep Kit	Illumina	Cat# RS-200-0012

**Deposited Data**		

Raw and analyzed data	This paper	GEO: GSE124546
ATAC-seq experiment	Lu et al., 2017	GEO: GSM2260231

**Experimental Models: Organisms/Strains**		

Col-0	N/A	N/A
*fwa-4*	Johnson et al., 2014	N/A
*fwa shh1*	This paper	N/A
*fwa clsy1*	This paper	N/A
*fwa nrpd1*	This paper	N/A
*fwa dcl3*	This paper	N/A
*fwa rdr1 rdr6*	This paper	N/A
*fwa rdr1 rdr2 rdr6*	This paper	N/A
*fwa suvh2 suvh9*	This paper	N/A
*fwa morc6*	This paper	N/A
*fwa rdm1*	This paper	N/A
*fwa dms3*	This paper	N/A
*fwa drd1*	This paper	N/A
*fwa ago4*	This paper	N/A
*fwa ago6 ago9*	This paper	N/A
*fwa ago4 ago6 ago9*	This paper	N/A
*fwa drm1 drm2*	This paper	N/A
*fwa cmt3*	This paper	N/A
*fwa rdr2*	Bond and Baulcombe, 2015	N/A
*fwa dcl2 dcl4*	Bond and Baulcombe, 2015	N/A
*fwa dcl2 dcl3 dcl4*	Bond and Baulcombe, 2015	N/A
*fwa nrpe1*	Bond and Baulcombe, 2015	N/A

**Recombinant DNA**		

pEG302-NRPD1-3xFLAG-ZF	This paper	N/A
pEG302- Hyg-NRPD1-3xFLAG-ZF	This paper	N/A
pEG302-RDR2-3xFLAG-ZF	This paper	N/A
pEG302-SHH1-3xMyc-ZF	This paper	N/A
pEG302-DMS3-3xFLAG-ZF	This paper	N/A
pEG302-Hyg-DMS3-3xFLAG-ZF	This paper	N/A
pEG302-RDM1-3xHA-ZF	This paper	N/A
pEG302-MORC6-3xHA-ZF	This paper	N/A
pEG302-MORC1-3xFLAG-ZF	This paper	N/A
pMDC123-ZF-3xFLAG-DRMcd	This paper	N/A
pEG302-3xHA-ZF	This paper	N/A
ZF-3xHA-SUVH9	Johnson et al., 2014	N/A
pMDC32-UBQ10::AP1hairpin	This paper	N/A
JP726	Johnson et al., 2008	N/A
JP726-Hyg	Rajakumara et al., 2011	N/A
pMDC123-UBQ10-3xFLAG-ZF	Gallego-Bartolomé et al., 2018	N/A
pMDC32	Curtis and Grossniklaus, 2003	N/A

**Software and Algorithms**		

BSMAP v2.74	Xi and Li., 2009	https://code.google.com/archive/p/bsmap/
Bowtie v1.0.0	Langmead et al., 2009	http://bowtie-bio.sourceforge.net/index.shtml
MACS2 v2.1.1	Zhang et al., 2008	https://pypi.org/project/MACS2/
NGSplot v2.41.4	Shen et al., 2014	https://github.com/shenlab-sinai/ngsplot
HOMER	Heinz et al., 2010	http://homer.ucsd.edu/homer/download.html
DMRcaller	Catoni et al., 2018	https://bioconductor.org/packages/release/bioc/html/DMRcaller.html
Tophat v2.0.13	Trapnell et al., 2009	https://ccb.jhu.edu/software/tophat/index.shtml
HTseq	Anders et al., 2015	https://pypi.org/project/HTSeq/
SAMtools v0.1.19	Li et al., 2009	http://samtools.sourceforge.net
DESeq	Anders and Huber, 2010	https://bioconductor.org/packages/release/bioc/html/DESeq.html
DESeq2	Love et al., 2014	https://bioconductor.org/packages/release/bioc/html/DESeq2.html

### Contact for Reagent and Resource Sharing

Further information and requests for resources and reagents should be directed to and will be fulfilled by the Lead Contact, Steven E. Jacobsen (jacobsen@ucla.edu)

### Experimental Model and Subject Details

The following mutants were introgressed in the *fwa-4* epiallele described in Johnson et al., 2014: *shh1-1* (SALK_074540C), *clsy1-7*(SALK_018319), *nrpd1-4*(SALK_083051), *dcl3-1*(SALK_005512), *rdr1-1*(SAIL_672_F11), *rdr2-1*(SAIL_1277_H08), *rdr6-15*(SAIL_617_H07), *morc6-3*(GABI_599B06), *suvh2*(SALK_079574), *suvh9*(SALK_048033), *rdm1-4* (EMS,(Gao et al., 2010), *dms3-4*(SALK_125019C), *drd1-6*(EMS,(Kanno et al., 2004)), *ago4-5*(EMS,(Greenberg et al., 2011)) for *fwa ago4* double mutant, *ago4-4*(FLAG_216G02) for *fwa ago4 ago6 ago9* quadruple mutant, *ago6-2* (SALK_031553), *ago9-1*(SALK_127358), *drm1-2*(SALK_031705), *drm2-2*(SALK_150863), *cmt3-11*(SALK_148381). The following mutants: *rdr2-2*(SALK_059661), *dcl2-1*(SALK_064627), *dcl4-2*(GABI_160G05), *dcl3-1*(SALK_005512) and *nrpe1-1*(EMS) were introgressed into *fwa-1* and described in Bond and Baulcombe, 2015.

All plants in this study were grown under long-day conditions (16h light/8h dark). Transgenic plants were obtained by agrobacterium-mediated floral dipping. T1 transgenic plants were selected on 1/2 MS medium + Glufosinate 50 μg/mL (Goldbio) or 1/2 MS medium + Hygromycin B 25 μg/mL (Invitrogen) in growth chambers under long day conditions and subsequently transferred to soil. Successive transgenic generations were germinated directly on soil. Flowering time was scored by counting the total number of rosette and caulinar leaves. In the flowering time dot plots, each dot represents the flowering time of individual plants. Plants with 20 or less leaves were considered early flowering. The same Col-0 and *fwa* controls were used for each T1 flowering time dotplot. The same *fwa, fwa nrpe1 and fwa drm1/2* controls were used for NRPD1-ZF and RDR2-ZF T2 flowering time dotplots. The same Col-0, *fwa, fwa nrpe1 and fwa drm1/2* controls were used for SHH1-ZF, DMS3-ZF and MORC1-ZF T2 flowering time dotplots. The same Col-0, *fwa, fwa nrpe1 and fwa drm1/2* controls were used for ZF-SUVH9 and MORC6-ZF T2 flowering time dotplots. To generate the DMS3-ZF x NRPD1-ZF co-targeting lines, homozygous lines expressing DMS3-ZF and ZF control in the Col-0 background were transformed with the pEG302-Hyg-NRPD1-3xFLAG-ZF construct described below. To generate the *AP1* RNA hairpin lines, homozygous lines expressing DMS3-ZF and ZF control in the Col-0 background were transformed with the *AP1* RNA hairpin construct described below. To score the *ap1* mutant phenotype, the average number of flowers per pedicel/peduncule present in the first 5 determinate structures in the main inflorescence following leaf production was calculated.

### Method Details

#### Plasmid Construction

##### NRPD1-3xFLAG-ZF

The ZF described in Johnson et al., 2014 was first cloned into the unique XhoI site of a modified pCR2 plasmid containing a 3xFLAG and a Biotin Ligase Recognition Peptide (BLRP) separated by a unique XhoI site. The fragment containing 3xFLAG-ZF-BLRP was digested with AscI and cloned into AscI-digested pENTR-NRPD1, which contains a genomic sequence of *NRPD1* (Law et al., 2011), to create pENTR-NRPD1-3xFLAG-ZF. The resulting plasmid was recombined into JP726 (Johnson et al., 2008) using LR clonase (Invitrogen) to create pEG302-NRPD1-3xFLAG-ZF. Also, the pENTR-NRPD1-3xFLAG-ZF plasmid was recombined using LR clonase into a modified version of JP726 containing a Hygromycin resistance cassette (JP726-Hyg) (Rajakumara et al., 2011), to create pEG302-Hyg-NRPD1-3xFLAG-ZF.

##### RDR2-3xFLAG-ZF

The same modified pCR2 plasmid described above containing 3xFLAG-ZF-BLRP was used to clone 3xFLAG-ZF-BLRP into AscI-digested pENTR-RDR2, that contains a genomic sequence of *RDR2* (Law et al., 2011), to create pENTR-RDR2-3xFLAG-ZF. The resulting plasmid was recombined into JP726 using LR clonase (Invitrogen) to create pEG302-RDR2-3xFLAG-ZF.

##### SHH1-3xMyc-ZF

The ZF was cloned into the unique XhoI site of the plasmid pENTR-SHH1-3xMyc-BLRP, which contains the genomic sequence of *SHH1* and a C-terminal 3xMyc-BLRP tag (Law et al., 2011) to create pENTR-SHH1-3xMyc-ZF. In this particular construction, a shorter ZF sequence with only five tandem copies of the ZF repeats was cloned instead of the six tandem copies present in ZF. The resulting plasmid was recombined into JP726 using LR clonase (Invitrogen) to create pEG302-SHH1-3xMyc-ZF.

##### DMS3-3xFLAG-ZF

The same modified pCR2 plasmid described above containing 3xFlag-ZF-BLRP was digested with AscI to clone 3xFLAG-ZF-BLRP into AscI-digested pEG302-DMS3, which contains a genomic sequence of *DMS3* (Law et al., 2010) to create pEG302-DMS3-3xFLAG-ZF. Also, the BASTA cassette in pEG302-DMS3-3xFLAG-ZF was replaced by the Hygromycin resistance cassette from JP726-Hyg to create pEG302-Hyg-DMS3-3xFLAG-ZF.

##### RDM1-3xHA-ZF

For this construct, a genomic sequence of RDM1 including 1584 base pairs of promoter sequence was cloned into the pENTR/D plasmid (Invitrogen) using the primers listed in Table S1 to create pENTR-RDM1. The ZF sequence was cloned into the unique XhoI site of a modified pCR2 plasmid containing 3xHA and BLRP separated by a unique XhoI site. The fragment containing 3xHA-ZF-BLRP was digested with AscI and cloned into AscI-digested pENTR-RDM1 to create pENTR-RDM1-3xHA-ZF. The resulting plasmid was recombined into JP726 using LR clonase (Invitrogen) to create pEG302-RDM1-3xHA-ZF.

##### MORC6-3xHA-ZF

The same modified pCR2 plasmid described above containing 3xHA-ZF-BLRP was digested with AscI to clone 3xHA-ZF-BLRP into an AscI-digested pENTR-MORC6 plasmid, which contains a genomic sequence of *MORC6* (Moissiard et al., 2014), to create pENTR-MORC6-3xHA-ZF. The resulting plasmid was recombined into JP726 using LR clonase (Invitrogen) to create pEG302-MORC6-3xHA-ZF.

##### MORC1-3xFLAG-ZF

The 3xFLAG-ZF-BLRP fragment in the modified pCR2 plasmid described above was digested with AscI and inserted in the single AscI site of the pENTR-MORC1 plasmid, which contains a genomic sequence of *MORC1* (Moissiard et al., 2014), to create pENTR-MORC1-3xFLAG-ZF. The resulting plasmid was recombined into JP726 using LR clonase (Invitrogen) to create pEG302-MORC1-3xFLAG-ZF.

##### ZF-3xFLAG-DRMcd

The pENTR-DRMcd plasmid containing the tobacco *DRM* catalytic domain (*DRMcd*), and described in Zhong et al., 2014 was recombined using LR clonase (Invitrogen) into pMDC123-UBQ10-3xFLAG-ZF (Gallego-Bartolomé et al., 2018), a modified pMDC123 plasmid (Curtis and Grossniklaus, 2003) containing the *Arabidopsis UBQ10* promoter followed by a BLRP-ZF-3xFLAG cassette located upstream of a gateway cassette, to create pMDC123-ZF-3xFLAG-DRMcd.

##### 3xHA-ZF

For this plasmid, the MORC6 coding region present in the pENTR-MORC6-3xHA-ZF plasmid described above was removed. First, StuI and ClaI restriction sites were introduced upstream and downstream of MORC6 coding sequence, respectively, by site-directed mutagenesis using the QuickChange II kit (Agilent). StuI-ClaI digested pENTR-MORC6-3xHA-ZF was treated with Klenow fragment (NEB) and re-ligated to create pENTR-pMORC6::3xHA-ZF. The resulting plasmid was recombined into JP726 using LR clonase (Invitrogen) to create pEG302-3xHA-ZF.

##### *AP1* RNA hairpin

For this construct we first cloned 1990bp of the *Arabidopsis UBQ10* promoter into HindIII/KpnI-digested pMDC32 plasmid (Curtis and Grossniklaus, 2003) to create pMDC32-UBQ10. A hairpin construct was designed based on the pHANNIBAL plasmid (Wesley et al., 2001), where a fragment containing 341bp of the *AP1* promoter (Chr1:25,986,324-25,986,664) and its 5′-3′ reverse complementary sequence were separated by 767bp of the *Pdk* intron and cloned into the KpnI/SpeI sites of pMDC32-UBQ10 plasmid to create pMDC32-UBQ10::AP1hairpin plasmid.

#### BS-PCR-seq

Leaf tissue from one adult plant of representative T2 lines was collected to analyze *FWA* methylation. Inflorescences from one plant of representative T2 lines were collected to analyze *AP1* methylation. DNA was extracted following a CTAB-based method and converted using the EZ DNA methylation-lighting kit (ZYMO research) or the Epitect Bisulfite Conversion kit (QIAGEN). For *FWA* promoter, we analyzed methylation over three different regions: Region 1 (chr4: 13038143-13038272); Region 2 (chr4: 13038356-13038499); Region3 (chr4: 13038568-13038695), which cover fragments of the promoter and 5′ transcribed region of *FWA*. For *AP1* promoter, we analyzed methylation over three different regions: Region 1: chr1: 25986270-25986391; Region 2: chr1: 25985976-25986098; Region 3: chr1: 25986235-25986108, which cover fragments of the promoter and 5′ transcribed region of *AP1*. To amplify the different regions, Pfu Turbo Cx (Agilent) was used together with primers containing the Illumina adaptors. The primers used are listed in Table S1. Different PCR products for the same sample were pooled and purified by AMPure beads (Beckman Coulter). Libraries were made from purified PCR products using a TruSeq Nano DNA Library Prep kit for Neoprep automated library preparation machine (Illumina), a Kapa DNA hyper kit (Kapa Biosystems) with Illumina TruSeq DNA adapters or a Ovation Ultralow V2 kit (NuGEN). Libraries were sequenced on Illumina HiSeq 2000 or HiSeq 2500.

#### Western Blot

Inflorescences from a pool of plants were ground in liquid nitrogen and proteins were extracted in IP buffer (50mM Tris pH 7.6, 150mM NaCl, 5mM MgCl2, 10% glycerol, 0.1% NP40, 0.5mM DTT, 10μM PMSF, 1.5μM pepstatin, 10μM MG132, complete-mini protease inhibitor EDTA-free (Roche)). After 3 centrifugation steps of 10 minutes at 10000 x g at 4°C, samples were resuspended in 1x Laemmli buffer and denatured at 95°C for 5 minutes. Antibodies against AGO4, AGO6 and AGO9 were used at 1:4000 and Actin C4 (MAB1501, Millipore) at 1:3000 in TBS-T. Fluorescently conjugated anti-mouse and anti-rabbit secondary antibodies (LI-COR) where used at 1:15000 in TBS-T. Proteins were visualized with an Odyssey CLx imaging system (LI-COR).

#### ChIP-seq

ChIPs were performed as described previously with minor modifications (Johnson et al., 2014). 2g of inflorescences were used for FLAG, HA, NRPE1 and AGO ChIPs. 2g of 12 day-old seedlings grown on 1/2 MS plates in a growth room in long days period were used for Pol II Ser5 ChIP. All samples were ground in liquid nitrogen and fixed for 10 minutes in Nuclei Isolation buffer containing 1% formaldehyde. After stopping the reaction with glycine, nuclei were isolated, chromatin was sheared using a Bioruptor Plus (Diagenode) and immunoprecipitated overnight at 4°C with Anti-FLAG M2 (Sigma), Anti-HA 3F10 (Roche), Anti-Pol II phospho Ser5 Ab5131 (Abcam), Anti-NRPE1 and NRPE1 preimmune serum for NRPE1 control (Liu et al., 2018), Anti-AGO4, Anti-AGO6 and Anti-AGO9 (provided by Dr Olivier Voinnet, ETH, Switzerland) antibodies. Chromatin-bound proteins were immunoprecipitated with magnetic Protein A and Protein G Dynabeads (Invitrogen) for 3h at 4°C, washed with Low Salt, High Salt, LiCl and TE buffers for 10 minutes at 4°C and eluted with elution buffer for 2x20 minutes at 65°C. Reverse crosslink was done overnight at 65°C, followed by proteinase K treatment at 45°C for 5h. DNA fragments were purified using phenol:chloroform and precipitated with NaAc/EtOH and GlycoBlue (Invitrogen) overnight at −20°C. Libraries were prepared using the Ovation Ultra Low System V2 1-16 kit (NuGEN) following the manufacturer’s instructions.

#### Whole Genome Bisulfite Sequencing (WGBS)

DNA from leaves of one adult plant grown on soil was extracted following a CTAB-based method. 100ng of DNA was sheared to 200bp with a Covaris S2 (Covaris) and used for library preparation using the Epitect Bisulfite Conversion kit (QIAGEN) and the Ovation Ultralow Methyl-seq kit (NuGEN) following the manufacturer’s instructions.

#### RNA-seq

For *FWA* expression in DMS3-ZF T2 lines (Figure S2C), RNA from pools of 12 day-old seedlings grown on 1/2 MS plates in long days period was extracted using Direct-zol kit (ZYMO research). For the rest of experiments, RNA from adult leaves grown on soil of one plant was extracted using Direct-zol kit (ZYMO research). 75ng of total RNA was used to prepare libraries using the Neoprep stranded mRNA-seq kit (Illumina). Alternatively, 1 μg of total RNA was used to prepare libraries using the TruSeq Stranded mRNA kit (Illumina).

#### sRNA-seq

RNA from inflorescences of one plant grown on soil was extracted using the Zymo Direct-zol Kit (ZYMO research). 2ug total RNA was run in 15% UREA gels and small RNAs from 15 to 30bp were cut and precipitated. This RNA was used to prepare libraries using the Truseq small RNA kit (Illumina) following manufacturer’s instructions.

#### ATAC-seq

For ATAC-seq in Col-0, raw data from previously published data (GSM2260231) (Lu et al., 2017) was used in this paper. Data was processed as described previously. ATAC-seq metaplots were plotted using NGSplot (v 2.41.4) (Shen et al., 2014).

### Quantification and Statistical Analysis

In order to test the significance of the difference between samples in Figures 5B, S5B, and S7B, Welch Two Sample t test with p < 0.05 was applied. Randomization/stratification/blinding were not applied to these experiments. No statistical calculation was used to estimate the sample size. Number of plants analyzed for flowering time experiments in Figures 1, 2, 3, S1, S2, and S3 is shown in Table S2. Number of plants analyzed for *ap1* mutant phenotype is shown in Figure S7G. Information about the number of plants and type of tissues used in the rest of experiments can be found in the Method Details section and in the following sections.

#### BS-PCR-seq

For BS-PCR-seq analysis, raw sequencing reads with designed BS-PCR primers were filtered followed by primer trimming with customized scripts. Trimmed reads were then aligned with BSMAP (v.2.74) (Xi and Li, 2009) to the reference TAIR10 genome by allowing up to 2 mismatches (-v 2), 1 best hit (-w 1) and aligning to both strands (-n 1). The methylation level at each cytosine was then extracted with BSMAP (methratio.py) scripts by allowing only unique mapped reads (-u). Reads with more than 3 consecutive methylated CHH sites were removed. Methylation levels at each cytosine were calculated as #C/(#C+#T). Cytosines with less than 20 reads coverage were discarded. To visualize the BS-PCR-seq data, only cytosines within designated regions were kept and plotted with customized R scripts. For representation of *AP1* BS-PCR-seq, the methylation levels present in the three regions analyzed were combined.

#### ChIP-seq

For ChIP-seq data analysis, raw reads were aligned to the *Arabidopsis* reference genome (TAIR10) with Bowtie (v1.0.0) (Langmead et al., 2009), allowing only uniquely mapping reads with fewer than two mismatches, and duplicated reads were removed with Samtools 0.1.19 (Li et al., 2009). For all ChIP-seq experiments, peaks were called using MACS2 (v 2.1.1.) (Zhang et al., 2008). Only FLAG or HA peaks with more than 2 fold of enrichment were used for following analysis. DMS3-ZF sites without NRPE1 recruitment correspond to ZF off-target sites with greater than 4 fold enrichment and FDR of 0.05, tested with R package DESeq (Anders and Huber, 2010), of FLAG DMS3-ZF ChIP-seq over NRPE1 ChIP-seq normalized readcounts. The rest of the ZF off-targets were considered sites with NRPE1 recruitment.

To call ChIP-seq peaks in Figure 4C, data from two independent lines of DMS3-ZF or ZF was pooled to increase the sequencing depth. In Figure 4G, FLAG and NRPE1 ChIP-seq data from two independent DMS3-ZF lines was pooled. For Figure 5A, NRPE1 ChIP-seq data from two independent DMS3-ZF lines was pooled.

Controls used in Figure 4D for FLAG ChIP-seq in DMS3-ZF lines and HA ChIP-seq in ZF lines were FLAG and HA ChIP-seq in Col-0, respectively. Control used in Figure 4G and Figure S7D for FLAG ChIP-seq in DMS3-ZF was FLAG ChIP-seq in Col-0. Control used in Figure 4G, S5B and S7D for NRPE1 ChIP-seq in DMS3-ZF was pre-immune serum ChIP-seq in DMS3-ZF. Control used in Figure S4H for FLAG and NRPE1 ChIP-seq in DMS3-ZF *fwa drm1/2* were FLAG and NRPE1 ChIP-seq in *fwa drm1/2,* respectively. Control used in Figure S4H for FLAG and NRPE1 ChIP-seq in DMS3-ZF *fwa ago4/6/9* were FLAG and NRPE1 ChIP-seq in *fwa ago4/6/9,* respectively. Controls used in Figure 6B for FLAG, NRPE1, AGO4 ChIP-seq in DMS3-ZF in *fwa* and *fwa nrpd1* backgrounds were FLAG, NRPE1 and AGO4 ChIP-seq in *fwa,* respectively.

Genomic distribution of ChIP-seq peaks was calculated using HOMER with default parameters (Heinz et al., 2010). In order to identify predominant motifs in ZF-associated ChIP-seq peaks, HOMER (Heinz et al., 2010) was applied to 200 bp around the ZF ChIP-seq peak summit. In order to select sites without ZF binding in Figure S5G, bedtools shuffle (v 2.24.0) were applied with ‘-chrom’ to kept random shuffled sites on the same chromosome as well as ‘-excl’ to exclude sites with ZF binding. ChIP-seq metaplots and heatmaps were generated using NGSplot (v 2.41.4) (Shen et al., 2014).

#### WGBS

For WGBS data analysis, raw reads were aligned to the reference TAIR10 genome using BSMAP (v 2.74) (Xi and Li, 2009) by allowing up to 2 mismatches (-v 2), 1 best hit (-w 1) and aligning to both strands(-n 1). Methylation levels at each cytosine were then extracted with BSMAP (methratio.py) scripts by allowing only unique mapped reads (-u). Reads with more than 3 consecutive methylated CHH sites were removed. Methylation levels at each cytosine were calculated as #C/(#C+#T). DMRs between DMS3-ZF and ZF, NRPD1-ZF and ZF, DMS3-ZF X NRPD1-ZF and ZF were calculated as before (Stroud et al., 2013). DMRs were then defined with R package DMRcaller (Catoni et al., 2018) as described before (Stroud et al., 2013) except that the 100bp window tested for DMR are 1kb flanking regions over the 10776 ZF binding sites. DMRs within 200bp of each other were merged for further analysis. For analysis of hyperDMRs in DMS3-ZF, NRPD1-ZF or DMS3-ZF X NRPD1-ZF, all three types of hyperDMRs (CG/CHG/CHH) were combined and merged if the distance between two DMRs were less than 200bp. Annotations of hyperDMR to the nearest genes were calculated using HOMER (Heinz et al., 2010). In order to call high confidence CHH hyperDMRs in DMS3-ZF *fwa nrpd1* (Figure 6B), after calling genome-wide DMRs, we filtered out DMRs with pre-existing CHH methylation greater than 0.05 as well as DMRs that overlapped with tRNA regions.

In Figures S1C and S2B, individual plants were used (n = 1). To call hyperDMRs in DMS3-ZF, NRPD1-ZF and DMS3-ZF X NRPD1-ZF, a control set was merged from four biological replicates of Col-0 (a biological replicate in this section consists of adult leaf tissue from one plant), two biological replicates from two independent T2 ZF lines, two biological replicates from two independent T3 ZF lines and one plant from two independent T3 ZF(-) lines (n = 14 in total). In Figure 5B, to call hyperDMRs in DMS3-ZF, data from two biological replicates from two independent T2 and T3 DMS3-ZF lines were pooled (n = 8 in total) and then compared to all the control set described above (n = 14). To plot heritable DNA methylation in Figure 5E, reads from one DMS3-ZF (-) plant from two independent DMS3-ZF lines (n = 2) were merged and plotted over all the reads pooled from the control set described above (n = 14). To call hyperDMRs in Figure 6B, data from one plant from two independent DMS3-ZF *fwa nrpd1* lines (n = 2) were pooled and compared to the signals merged from two biological replicates of *fwa nrpd1* (n = 2). To call hyperDMRs in DMS3-ZF X NRPD1-ZF in Figure 7C, one plant from three independent T1 lines generated from NRPD1-ZF transformation into homozygous DMS3-ZF line 1 and one plant from three independent T1 lines generated from NRPD1-ZF transformation into homozygous DMS3-ZF line 2 (n = 6), were pooled and compared to the control set described above (n = 14). To call hyperDMRs in NRPD1-ZF in Figure 7C, one plant from three independent T1 lines generated from NRPD1-ZF transformation into homozygous ZF line 1 and one plant from three independent T1 lines generated from NRPD1-ZF transformation into homozygous ZF line 2, as well as one plant from two independent T1 lines of NRPD1-ZF transformed in Col-0 were pooled (n = 8 in total) and compared to the control set described above (n = 14).

#### RNA-seq

For RNA-seq data analysis, raw reads were first aligned to TAIR10 gene annotation using Tophat (v 2.0.13) (Trapnell et al., 2009) by allowing up to two mismatches and only allowing one multiple hits. When reads did not map to the annotated genes, the reads were mapped to the TAIR10 genome. Number of reads mapping to genes were calculated by HTseq (Anders et al., 2015) with default parameters. Expression levels were determined by RPKM (reads per kilobase of exons per million aligned reads). Differentially expressed genes were defined with R package DESeq (Anders and Huber, 2010) using a 2 fold change and FDR less than 0.05 as cut off.

For RNA-seq used in Figure 5C, three biological replicates (a biological replicate in this section consists of adult leaf tissue from one plant) from two independent DMS3-ZF T3 lines (n = 6), and three biological replicates from three independent ZF T3 lines (n = 9) were used. For RNA-seq used in Figure 7D, one plant from four independent DMS3-ZF X NRPD1-ZF T1 lines (n = 4), and three biological replicates from two independent ZF lines (n = 6) were used. For NRPD1-ZF RNA-seq in Figure 7G, one plant from three independent NRPD1-ZF T1 lines (n = 3), and three biological replicates from two independent ZF lines (n = 6) were used. For RNA-seq analysis in Figures 5C and 7D, individual replicates were applied for DESeq (Anders and Huber, 2010).

#### sRNA-seq

For sRNA-seq data analysis, raw reads were trimmed for Illumina adaptors using Cutadapt (v 1.9.1) and mapped to the TAIR10 reference genome using Bowtie (v1.1.0) (Langmead et al., 2009) allowing only one unique hit (-m 1) and zero mismatch. In order to define off-target sites with 24-nt siRNAs production, 1kb flanking regions of NRPE1-containing off-target regions were first divided into 100bp bins. Then 24-nt siRNAs count were calculated over those 100bp bins and DEseq2 (Love et al., 2014) was applied using 4 fold change and FDR less than 0.05 as cut off for DMS3-ZF versus ZF, NRPD1-ZF versus ZF and DMS3-ZF X NRPD1-ZF versus ZF.

To call regions with 24-nt siRNA enrichment in Figure 5A, three biological replicates (a biological replicate in this section consists of inflorescence tissue from one plant) from two independent DMS3-ZF lines (n = 6), and three biological replicates from two independent ZF lines (n = 6) were consider as individual replicates for DESeq2 (Love et al., 2014). For metaplot and heatmap of 24-nt siRNA in Figure S5C, three biological replicates from two independent DMS3-ZF lines (n = 6), and three biological replicates from two independent ZF lines (n = 6) were pooled. For Figure S5C, control used for sRNA-seq in DMS3-ZF was sRNA-seq in ZF. For metaplots and heatmaps of sRNA-seq in Figure 6B, one representative replicate for the indicated lines is shown. Controls used for Figure 6B sRNA-seq in DMS3-ZF in *fwa* and *fwa nrpd1* were sRNA-seq in untransformed *fwa* and *fwa nrpd1,* respectively. For sRNA-seq in Figure 7B, one plant from three independent NRPD1-ZF T1 lines (n = 3) and one plant from two independent DMS3-ZF X NRPD1-ZF T1 lines were applied for DESeq2 (Love et al., 2014).

### Data and Software Availability

#### Accession codes

The accession number for all the high-throughput sequencing data reported in this paper is GEO: GSE124546.

## Supplemental Information

Table S1. Primers, Related to STAR Methods

Table S2. Number of Plants Analyzed in Flowering Time Experiments, Related to STAR Methods
