# Supplementary material for: Co-targeting RNA Polymerases IV and V Promotes Efficient De Novo DNA Methylation in Arabidopsis
Source: Cell. 2019 Feb 21;176(5):1068–1082.e19. doi: 10.1016/j.cell.2019.01.029 (PMC6386582; doi:10.1016/j.cell.2019.01.029)
Supplement: Table S1. Primers, Related to STAR Methods [file mmc1.pdf]

Cell, Volume *176*

Supplemental Information

Co-targeting RNA Polymerases IV and V Promotes Efficient *De Novo* DNA Methylation in *Arabidopsis*

Javier Gallego-Bartolomé, Wanlu Liu, Peggy Hsuanyu Kuo, Suhua Feng, Basudev Ghoshal, Jason Gardiner, Jenny Miao-Chi Zhao, Soo Young Park, Joanne Chory, and Steven E. Jacobsen

Table S1: Primers. Related to STAR methods

	Use	Sequence 5´- 3´
***RDM1* FWD**	Clone genomic *RDM1*	CACCATCATGGTATTGTAGACTAAAAC
***RDM1* REV**	Clone genomic *RDM1*	TTTCTCAGGAAAGATTGGGTCAATG
***FWA* region 1 FWD**	BS-PCR-seq *FWA* region 1	TCATATAAAAAAAAAATTAAATTTCATTTCACAATAACCATT
***FWA* region 1 REV**	BS-PCR-seq *FWA* region 1	GTATGGGYTTYGATAAAGAATATATGAGATTYT
***FWA* region 2 FWD**	BS-PCR-seq *FWA* region 2	CTCATATATACCTTATCCCATTCAACATTCATA
***FWA* region 2 REV**	BS-PCR-seq *FWA* region 2	AAGATYTGATATTTGGYTGGAAAAAAYAATAATAAT
***FWA* region 3 FWD**	BS-PCR-seq *FWA* region 3	CRCTCTTTATCCCATTCAACATTCATAC
***FWA* region 3 REV**	BS-PCR-seq *FWA* region 3	TTTGGTTGAAAAAAATAATAAAAATTTGATTGTYAGTAT
***AP1* region 1 FWD**	BS-PCR-seq *AP1* region 1	GTTTTATTAAAAAATTATGGATYYGATATTAGTAYGAGATATA
***AP1* region 1 REV**	BS-PCR-seq *AP1* region 1	TTTTTCTTTCTTTCCTTTATAAAAAAACTTATATTTAAATCTC
***AP1* region 2 FWD**	BS-PCR-seq *AP1* region 2	TTTAGAGTAAGAAGTTTTTTAAAAAAGGATTAAAAATGG
***AP1* region 2 REV**	BS-PCR-seq *AP1* region 2	AAAAATTTTCCCTTATAAAAAAAAAACAACAAAAACAA
***AP1* region 3 FWD**	BS-PCR-seq *AP1* region 3	AAAGATTYYGAGATTTAAATATAAGTTTTTTTATAAAGGAAAG
***AP1* region 3 REV**	BS-PCR-seq *AP1* region 3	ACCCTACCCCTTCCCATTTTTA
