# Supplementary material for: Co-targeting RNA Polymerases IV and V Promotes Efficient De Novo DNA Methylation in Arabidopsis
Source: Cell. 2019 Feb 21;176(5):1068–1082.e19. doi: 10.1016/j.cell.2019.01.029 (PMC6386582; doi:10.1016/j.cell.2019.01.029)
Supplement: Table S2. Number of Plants Analyzed in Flowering Time Experiments, Related to STAR Methods [file mmc2.pdf]

Table S2. Number of plants analyzed in flowering time experiments. Related to STAR methods

Number of T1 plants shown in the flowering time dotplots in Figures S1, S2, S3

The same controls were used for all T1 flowering time dotplots.

	background	n plants
**Controls**	Col-0	47
	fwa	46
**NRPD1-ZF**	**background (in *fwa* x)**	**n plants**
	ago4.	47
	ago4/6	93
	ago4/6/9	43
	clsy1	38
	cmt3	58
	dcl3	40
	dcl2/4	55
	dcl2/3/4	41
	drm1/2	40
	nrpd1	47
	nrpe1	62
	rdr2	50
	shh1	41
	wt	47
**RDR2-ZF**	**background (in *fwa* x)**	**n plants**
	clsy1	113
	drm1/2	70
	nrpd1	71
	nrpe1	47
	rdr2	40
	shh1	48
	wt	42
**SHH1-ZF**	**background (in *fwa* x)**	**n plants**
	clsy1	49
	drm1/2	55
	nrpd1	114
	nrpe1	53
	rdr2	76
	shh1	45
	wt	44
**DMS3-ZF**	**background (in *fwa* x)**	**n plants**
	ago4.	55
	ago4/6	62
	ago4/6/9	96
	cmt3	60
	dms3	41
	drd1	74
	drm1/2	96
	morc6	46
	nrpd1	53
	nrpe1	65
	rdm1	72
	rdr1/6	63
	rdr1/2/6	45
	suvh2/9	39

	wt	60
**RDM1-ZF**	**background (in *fwa* x)**	**n plants**
	dms3	95
	drd1	62
	drm1/2	79
	morc6	57
	nrpd1	108
	nrpe1	60
	rdm1	54
	suvh2/9	70
	wt	82
**ZF-SUVH9**	**background (in *fwa* x)**	**n plants**
	dms3	61
	drd1	35
	drm1/2	65
	morc6	79
	nrpd1	58
	nrpe1	73
	rdm1	94
	suvh2/9	42
	wt	49
**MORC6-ZF**	**background (in *fwa* x)**	**n plants**
	dms3	75
	drd1	48
	drm1/2	81
	morc6	21
	nrpd1	138
	nrpe1	58
	rdm1	56
	suvh2/9	41
	wt	47
**MORC1-ZF**	**background (in *fwa* x)**	**n plants**
	morc6	39
	wt	46
**ZF-DRMcd**	**background (in *fwa* x)**	**n plants**
	cmt3	55
	drm1/2	47
	nrpd1	47
	nrpe1	75
	wt	106

Number of T2 plants shown in the flowering time dotplots in Figures 1, 2, 3 and S3

The same controls were used for NRPD1-ZF and RDR2-ZF dotplots except Col-0.

The same controls were used for SHH1-ZF, DMS3-ZF and MORC1-ZF dotplots.

The same controls were used for ZF-SUVH9 and MORC6-ZF dotplots.

				**n plants**			
**controls**		**NRPD1-ZF**	**background (in *fwa x)***	**line 1**	**line 2**	**line 3**	**line 4**
Col-0	16		*ago4.*	17	15	15	17
fwa	16		*ago4/6*	14	14	8	14
fwa drm1/2	16		*ago4/6/9*	11	12	10	12
fwa nrpe1	14		*clsy1*	19	18	18	15
			cmt3	15	19	18	16
			dcl3	16	17	20	16

			dcl2/4	21	17	18	16
			dcl2/3/4	26	27	12	29
			drm1/2	11	15	17	20
			nrpd1	19	19	18	19
			nrpe1	14	15	14	14
			rdr2	16	14	18	15
			shh1	19	11	20	10
			wt	21	16	20	14

				**n plants**			
**controls**		**RDR2-ZF**	**background (in *fwa* x)**	**line 1**	**line 2**	**line 3**	**line 4**
Col-0	14		*clsy1*	17	15	15	17
fwa	16		*drm1/2*	16	16	16	15
fwa drm1/2	16		*nrpd1*	15	15	15	15
fwa nrpe1	14		*nrpe1*	15	14	14	14
			rdr2	16	18	17	11
			shh1	7	11	13	11
			wt	13	14	15	14

				**n plants**			
**controls**		**SHH1-ZF**	**background (in *fwa* x)**	**line 1**	**line 2**	**line 3**	**line 4**
Col-0	10		*clsy1*	12	16	14	13
fwa	12		*drm1/2*	17	14	16	15
fwa drm1/2	11		*nrpd1*	14	13	12	12
fwa nrpe1	11		*nrpe1*	13	14	12	11
			rdr2	12	13	15	14
			shh1	13	16	12	18
			wt	14	17	13	16

				**n plants**			
**controls**		**DMS3-ZF**	**background (in *fwa* x)**	**line 1**	**line 2**	**line 3**	**line 4**
Col-0	10		*ago4.*	10	15	14	15
fwa	12		*ago4/6*	12	13	16	19
fwa drm1/2	11		*ago4/6/9*	12	34	35	16
fwa nrpe1	11		*cmt3*	15	15	17	13
			dms3	15	18	17	14
			drd1	15	14	12	10
			drm1/2	35	33	18	15
			morc6	12	14	15	16
			nrpd1	20	19	18	17
			nrpe1	11	10	13	13
			rdm1	34	16	14	35
			rdr1/6	16	14	15	12
			rdr1/2/6	6	12	11	12
			suvh2/9	14	16	19	9
			wt	16	18	15	16

				**n plants**			
**controls**		**RDM1-ZF**	**background (in *fwa* x)**	**line 1**	**line 2**	**line 3**	**line 4**
Col-0	17		*dms3*	15	14	14	14
fwa	16		*drd1*	12	11	13	15
fwa drm1/2	13		*drm1/2*	18	16	15	15
fwa nrpe1	13		*morc6*	17	14	15	18
			nrpd1	19	17	15	13
			nrpe1	16	16	15	15
			rdm1	18	18	13	20
			suvh2/9	21	16	15	18
			wt	20	12	16	15

						n plants			
**controls**		**ZF-SUVH9**	**background**	**(in *fwa* x)**	**line 1**	**line 2**	**line 3**	**line 4**
Col-0	10		*dms3*		16	17	17	17
fwa	13		*drd1*		18	19	16	14
fwa drm1/2	10		*drm1/2*		17	17	18	16
fwa nrpe1	11		*morc6*		20	20	16	16
			nrpd1		12	13	12	13
			nrpe1		9	9	9	10
			rdm1		15	16	13	15
			suvh2/9		13	17	18	22
			wt		21	14	21	18
					**n plants**			
**controls**		**MORC6-ZF**	**background**	**(in *fwa* x)**	**line 1**	**line 2**	**line 3**	**line 4**
Col-0	10		*dms3*		19	17	16	18
fwa	13		*drd1*		17	17	15	13
fwa drm1/2	10		*drm1/2*		17	16	15	17
fwa nrpe1	11		*morc6*		19	17	15	15
			nrpd1		13	12	9	10
			nrpe1		17	16	16	16
			rdm1		17	17	15	14
			suvh2/9		18	19	20	17
			wt		16	19	20	18
					**n plants**			
**controls**		**MORC1-ZF**	**background**	**(in *fwa* x)**	**line 1**	**line 2**	**line 3**	**line 4**
Col-0	10		*morc6*		14	14	16	16
fwa	12		*wt*		17	14	14	15
fwa drm1/2	11							
fwa nrpe1	11							
					**n plants**			
**controls**		**ZF-DRMcd**	**background**	**(in *fwa* x)**	**line 1**	**line 2**	**line 3**	**line 4**
Col-0	15		*cmt3*		17	12	11	13
fwa	11		*drm1/2*		14	15	13	12
fwa drm1/2	14		*nrpd1*		13	16	13	16
fwa nrpe1	15		*nrpe1*		15	13	12	13
			wt		14	15	17	16

Number of T3 plants shown in the flowering time dotplots in Figure S1F

	n plants
Col-0	39
fwa	12
NRPD1-ZF (+)	13
NRPD1-ZF (-)	16
